# MultiStageSearch: An Iterative Workflow for Unbiased Taxonomic Analysis of Pathogens Using Proteogenomics

**DOI:** 10.1021/acs.jproteome.4c00901

**Published:** 2025-05-19

**Authors:** Julian Pipart, Tanja Holstein, Lennart Martens, Thilo Muth

**Affiliations:** † Data Competence Center MF 2, 9222 Robert Koch Institute, Berlin 13353, Germany; ‡ CompOmics, 54498 VIB Center for Medical Biotechnology, VIB, Ghent 9000, Belgium; § Department of Biomolecular Medicine, Faculty of Medicine and Health Sciences, Ghent University, Ghent 9000, Belgium; ∥ BioOrganic Mass Spectrometry Laboratory (LSMBO), IPHC UMR 7178, University of Strasbourg, CNRS, Strasbourg 67000, France; ⊥ Infrastructure Nationale de Protéomique ProFIFR2048, Strasbourg 67087, France

**Keywords:** SARS-CoV-2, peptide-spectrum matches (PSMs), open reading frame (ORF), “Norovirus GII”, Reverse Transcription-Polymerase Chain Reaction (RT-PCR)

## Abstract

The global SARS-CoV-2 pandemic emphasized the need for accurate pathogen diagnostics. While genomics is the gold standard, integrating mass spectrometry-based proteomics offers additional benefits. However, current proteomic and genomic reference databases are often biased toward specific taxa, such as pathogenic strains or model organisms, and proteomic databases are less comprehensive. These biases and gaps can lead to inaccurate identifications. To address these issues, we introduce MultiStageSearch, a multistep database search method that combines proteome and genome databases for taxonomic analysis. Initially, a generalist proteome database is used to infer potential species. Then, MultiStageSearch generates a specialized proteogenomic database for precise identification. This database is preprocessed to filter duplicates and cluster identical open reading frames to reduce genomic database biases. The workflow operates independently of strain-level NCBI taxonomy, enabling the identification of strains not represented in existing taxonomies. We benchmarked the workflow on viral and bacterial samples, demonstrating its superior performance in strain-level taxonomic inference compared to existing methods. MultiStageSearch offers a flexible and accurate approach for pathogen research and diagnostics, overcoming incomplete search spaces and biases inherent in reference databases.

## Graphical abstract

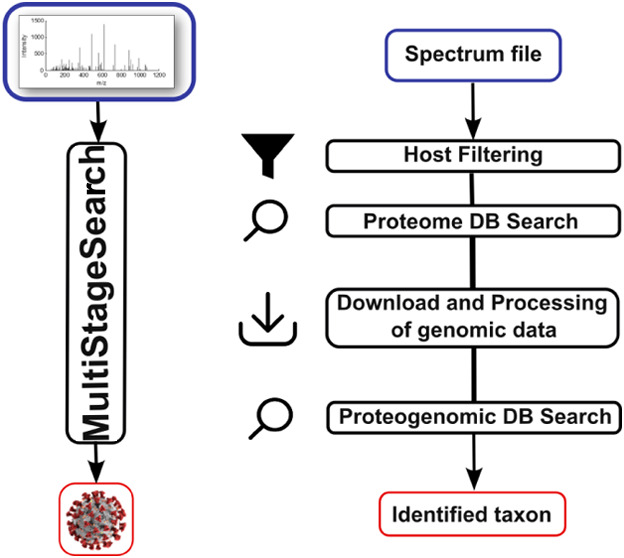

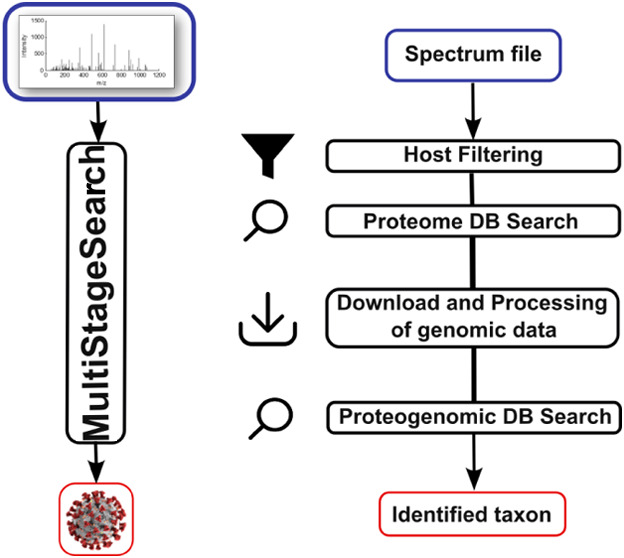

## Introduction

Viral and bacterial pathogens represent significant threats to public health,^1^ as evidenced by the recent SARS-CoV-2 pandemic. In suspected cases of outbreaks, the availability of fast and accurate detection and identification methods for these pathogens is essential to facilitate timely intervention and containment measures.^2^ Routinely used diagnostic methods, such as Reverse Transcription-Polymerase Chain Reaction (RT-PCR),^3^ have the disadvantage of requiring prior knowledge of the target pathogens.^4^ Consequently, for applications in diagnostics and the detection of novel or uncommon pathogens, open-view approaches such as next-generation sequencing genomics and mass spectrometry-based proteomics come into play.^5^ What sets proteomics apart from other methods is its unparalleled ability to deliver both qualitative and quantitative insights into virus-specific proteins with high accuracy, precision, and reproducibility. This capability has been particularly evident in SARS-CoV-2 studies, showcasing advancements in areas such as phosphoproteomics^6^ and affinity-purification for protein interaction mapping.^7^ While genomics has established itself as the gold-standard method for viral surveillance,^8^ various studies have demonstrated the advantages of proteomics as an orthogonal approach for viral^9,10^ and bacterial^11–14^ identification. In addition, MS-based identification approaches excel at delivering protein-level findings, such as information on antibiotic resistance markers.^15^ Pathogen diagnostics based on MS measurements can be performed in targeted or nontargeted modes, or a combination of both, at both the MS1 ^16–18^ and MS2 ^19^ levels. For a comprehensive overview on MS-based pathogen proteotyping methods, refer to Grenga et al.^20^ With continuous advances in instrumentation, tandem mass-spectrometry preceded by liquid chromatography (LC-MS/MS) offers high resolution at both the peptide and protein levels^21^ and has been successfully used for a quantitative assay targeting SARS-CoV-2 proteins.^9^

The standard downstream bioinformatic analysis of proteomic samples relies on searching the MS/MS spectra against proteomic reference databases.^22^ Various specific algorithms and software tools have been developed for the identification of taxon-unique peptides in bacterial, viral, and microbial community samples, including, among others, BACid,^23,24^ Pipasic,^25^ TCUP,^26^ MiCiD,^27,28^ PepGM,^29^ and further methods.^20,30^ These methods commonly rely on database searches against multispecies proteome databases, which often focus on specific pathogens depending on the application. However, searching against proteome databases poses challenges due to the limited number of available reference proteomes and inherent biases, particularly toward model organisms. Various versions of comprehensive reference databases exist, and many are available online. For databases such as NCBI^31^ and Uniprot,^32^ there are curated subsets, where the proteomic references and their taxonomic assignments have passed a quality control.^33,34^ However, the curated subsets can have a limited range regarding their representation of protein sequence and taxonomic diversity, especially when investigating organisms less common for proteomic applications, such as in viral or microbiome studies.^35–37^ It may therefore be beneficial to include all available proteomes, including uncurated ones, to achieve a more comprehensive taxonomic analysis. Here, we define “uncurated” as any protein or genome sequence uploaded by researchers to repositories such as GenBank.^38^ While these sequences must adhere to formatting guidelines, they have not undergone external validation.

To make best use of all the proteome information available, workflows using a two-step search approach have been developed.^39^ Jagtap et al. propose using primary search results from a large database to create a smaller subset for a subsequent search against a target-decoy version merged with a host database. This has been primarily been adopted in metaproteomics with large search spaces. As another related approach of iterative searching, Kuhring et al. use a first proteomic database search against a database with low taxonomic resolution but a broad representation across possibly present species, to identify potentially present taxa (or proteins).^40^ This is followed by a second search against all higher-resolved proteomes available for appropriate candidate species. While this strategy has shown to be successful for many samples, it can also lead to incorrect taxonomic assignments. Previous studies^29^ have suggested that this may result from biases present in the reference databases. In fact, biases toward model organisms in both genome and proteome databases have been well-documented.^41^ Researchers are encouraged to upload proteomes to databases such as UniProtKB or NCBI, which broadens the representation of reference proteins and increases taxonomic diversity by incorporating more species, strains, and isolates. However, this practice can inadvertently introduce substantial biases, as strains or species that are, for example, easily available through distributors, or commonly infect humans,^42^ such as certain Influenza strains^43^ tend to become overrepresented. This overrepresentation of proteins can increase the likelihood of identifying spectra associated with them, in turn biasing taxonomic analysis results based on these identifications. These reference-related biases, initially described in genomics,^44,45^ are equally relevant to proteome reference databases.

Several potential solutions have been proposed to mitigate the effects of reference bias, including the clustering of overly similar references to reduce overrepresentation.^46^ However, a different approach to address database bias while leveraging a maximum of available references is to use genome databases. Even though genome databases may also harbor biases, genomics is a more established field for analyzing viral samples, leading to a more comprehensive representation of viral protein, strain, and species diversity, which can enhance the robustness of taxonomic analyses.^47^

To address the limitations of bias in proteome reference databases and make use of additional genomic information, we introduce MultiStageSearch, a multistep proteogenomic workflow designed for strain-level identification of viral and bacterial samples. In a first step, a traditional database search is performed against a generalist proteome reference database. Based on the resulting initial search matches, potential candidate taxa are identified, and a proteogenomic database is constructed for these taxa using six reading-frame translation. Tailored strategies are used to identify, filter, and download appropriate reference genomes for database construction. This approach aims to provide taxonomic assignments for single organism samples, overcoming representation bias in both proteome and genome databases and enhancing the accuracy of strain-level identifications.

MultiStageSearch, written in Python and implemented as a Snakemake workflow, is freely available and open source under the MIT license at https://github.com/rki-mf2/MultiStageSearch.

## Methods

### Workflow Overview

Here, we introduce the MultiStageSearch workflow, providing a detailed description of each search step. MultiStageSearch performs a total of up to four database search steps to achieve strain-level identification results for experimental MS/MS spectra. Throughout the workflow, strain-level genomes are automatically queried and downloaded from the NCBI nucleotide database.^48^ Two distinct approaches are then used to perform a proteogenomic database search.

The input to MultiStageSearch includes an MGF file containing the MS spectra, a parameter file for the database search using SearchGUI,^49^ a reference database (e.g., RefSeqViral^50^), a file mapping protein accession numbers to taxonomic identifiers (IDs), and optionally, a host and common contaminants (https://www.thegpm.org/crap/) database.

Each search step (Host Filtering, Reference DB Search, Genomic DB Search, Top-Scoring DB Search) uses SearchGUI^49^ to add decoys to the provided database for the target-decoy search approach.^51^ Subsequently, SearchGUI is called to perform the database search using X!Tandem.^52^ Postprocessing is either performed by PeptideShaker,^53^ or by MS2Rescore.^54^ Because MS2Rescore requires additional configuration for each sample, including the regular expression to match the spectrum titles, it needs to be set up for each sample individually. When choosing PeptideShaker, however, MultiStageSearch is able to perform the database search for multiple samples simultaneously.

The complete MultiStageSearch workflow is built in Snakemake^55^ and can be broadly divided into the steps shown in Figure 1. The code has been tested with Python version 3.9. Computations were performed on Linux compute servers at the FU Berlin. Additional packages used in this workflow are summarized in Table S1 in the Supporting Information. The detailed workflow steps are described in the following sections.

**Figure 1:**
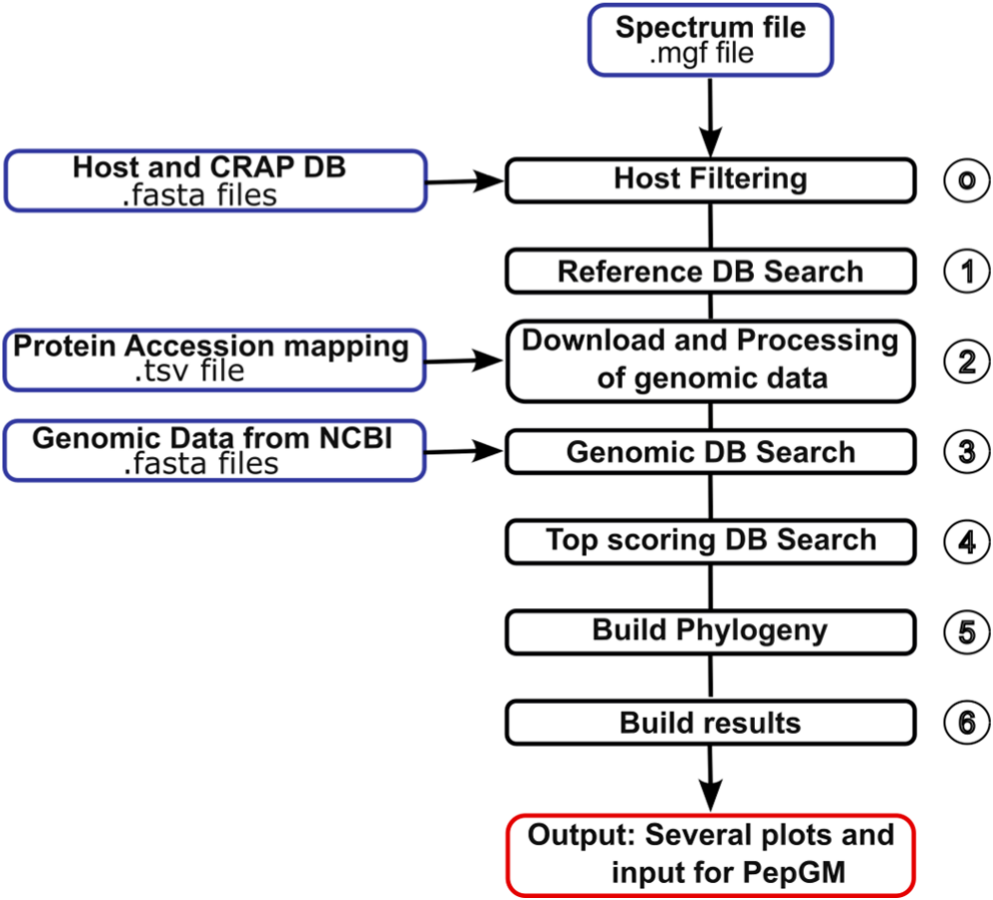
Overview of the MultiStageSearch workflow. Blue boxes indicate workflow inputs. Black boxes represent main workflow steps. The red box summarizes workflow output.

### Host Filtering

The user has the option to filter the experimental spectra, provided in MGF format, against a specified host and contaminant database. After searching and identifying peptide-spectrum matches (PSMs), these matches are removed from the original MGF file. This Host Filtering step is optional and is enabled by default. If Host Filtering is disabled, the Reference DB Search is conducted on the original MGF file.

### Reference DB Search

MultiStageSearch then performs a database search against the user-provided fasta reference database, as described above. It is recommended to use a reference database covering a broad range of viral or bacterial species, such as RefSeqViral for viruses or RefSeqBacterial^50^ for bacteria. If there is prior knowledge about the sample, such as the host species or potential host symptoms, the database can be tailored accordingly. PSMs are imported into MS2Rescore or PeptideShaker for postprocessing, rescoring, and results export.

The resulting output of PeptideShaker or MS2Rescore contains a list of PSMs, which will serve as the basis for inference of the appropriate candidate species to be used in the downstream workflow. To this end, MultiStageSearch uses a weighted aggregation of PSM counts per taxon, similarly to those described in previous studies.^29,40^ The weight of a PSM is based on its degeneracy: the more proteins a PSM can be attributed to, the lower its weight. To account for this, the weight of a PSM is calculated using the following formula:

1 $$\mathrm{PSM}\text{\_}{\mathrm{weight}}_{i}=\frac{1}{\sum \mathrm{proteins}\left({\mathrm{PSM}}_{i}\right)}$$

As shown in eq 1 the weight is inversely proportional to the number of proteins associated with a given PSM. Once weighted, these PSMs are then mapped to the corresponding species’ taxon IDs, assuming that the proteins contain NCBI identifiers, as in the case with RefSeqViral. The weights for each taxon ID are summed and written to a TSV file, which serves as input for the next step. The user-defined heuristic, “max_weight_differences”, determines how many taxa will be used to identify candidate strains for the next analysis steps. Often, the weight differences between taxon IDs are high, so this threshold ensures that MultiStageSearch considers only the necessary number of taxon IDs. The identified candidate taxa are then used as input for the next workflow step.

### Download and Processing of Genomic Data

#### Download of Candidate Strain Genomes

In this step, strain-level genomes for the top-scoring species-level taxon IDs are automatically queried from the NCBI database, taking into account all genome references available in NCBI. However, several challenges arise when downloading the appropriate genome sequences, which are addressed using situation-specific solutions as explained below to enhance the robustness and replicability of the workflow. Moreover, several user-adjustable parameters help define and refine the query.

- number_of_taxids: Defines the maximum number of species taxa to take into account for the query.
- max_weight_differences: Defines the maximum weight difference between the last taxon ID considered by the workflow and the next top-scoring taxon ID. If the difference is too great, the next taxon ID will not be considered. During the development of MultiStageSearch, the recommended value of 2 was chosen empirically.
- max_number_accessions: Defines the maximum number of genbank^38^ accession numbers that will be processed.
- sequence_length_diff: Defines the maximum sequence length difference between the last sequence considered by the workflow and the next longest sequence. If the difference is to great, the next sequence will not be considered.
- max_sequence_length: Defines the maximum sequence length. This can be used to avoid whole genome shotgun sequence entries.
- use_NCBI_Taxa: Uses the NCBI Taxonomy^31^ for the query.
- only_use_complete_genomes: Searches only for sequences with “complete” in the title (e.g., “complete genome”).

In the NCBI database, genomes are often not linked correctly to the corresponding taxon ID of the strain, but to the species. Therefore, MultiStageSearch uses the title of the database entry instead of the taxon ID when using the NCBI Taxonomy. If use_NCBI_Taxa is set to “True”, MultiStageSearch first finds all (alternative) names for the species. Afterward, all combinations of species name and corresponding strain name from the NCBI taxonomy are queried and the sequences matching the parameters are downloaded. If use_NCBI_Taxa is set to “False”, MultiStageSearch will search for sequence entries with the taxon ID of the species. This approach is able to find more entries, and also retrieves strains not present in the NCBI taxonomy.

Based on the information in the retrieved database entries, a single TSV file is generated, containing the values genbank_accession, species, strain, isolate, taxa, and HigherTaxa for the downloaded sequences. Taxa and HigherTaxa correspond to the strain taxon ID, and species taxon ID, respectively. While the strain taxon ID can be inferred when using the “use_NCBI_Taxa” option, there are no taxon IDs available for most strains when the “use_NCBI_Taxa” option is set to “false”. Therefore, in those cases, MultiStageSearch assigns custom taxon IDs to each downloaded sequence, starting at 5.000.000 to avoid overlap with existing taxon IDs.

#### Generation of the Proteogenomic Reference Database

For the subsequent database search step, proteome reference databases are required. Sixpack^56^ performs a six-frame translation of nucleotide sequences proteomes, which are then concatenated for further processing.

However, since the download process often retrieves multiple genomes—and thus multiple proteomes—for the same strain, a filtering method is implemented to eliminate duplicate proteomes post-translation. This method compares the combination of strain and isolate names extracted from the TSV file generated during genome download. If names match, the proteomes are compared by evaluating each open reading frame (ORF) of proteome *P*_1_ against each ORF of proteome *P*_2_. The similarity between two proteomes is computed as follows:

2 $$similarity_{P_{1},P_{2}}=\frac{\sum \;identical\;ORFs}{\max\left(len\left(P_{1}\right),\mathrm{len}\left(P_{2}\right)\right)}$$

Two ORFs are considered identical if their sequences match entirely, and the number of ORFs it contains is used as a proxy for proteome length. If a user-defined similarity threshold (default 98%) is reached, only the longer proteome among these similar ones is retained for further analysis. As shown in eq 2 the similarity measure accounts for the number of identical ORFs relative to the larger of the two proteomes, ensuring a normalized comparison. Subsequently, SearchGUI is used to generate decoys for the resulting proteome database. Despite the efforts to reduce redundancy, the database may still contain numerous identical ORFs due to genomes corresponding to strains of the same parent species. To address this issue, identical ORFs are clustered to reduce database, and thus search space, size. If ORFs share the same sequence, their sequence headers are combined to maintain full sequence provenance, while the sequence is stored only once. Moreover, to facilitate downstream ORF mapping to strains, a mapping file is created using an approach similar to that employed for the reference database search.

### Genomic DB Search

The downloaded and processed strain genomes form the basis of the subsequent database search. Given the more established nature of genomics compared to proteomics,^57^ typically a greater number of genomes, and thus sequences, are available at the strain level using this proteogenomic approach. This in turn increases the number of PSMs obtained. Similar to the first search step, the resulting PSMs from the proteogenomic database search are then weighted and aggregated by taxon ID using the same weighting applied in the initial search step. The outcome is a list of top-scoring, strain-level taxa identified through the workflow.

### Build Phylogeny

Optionally, a phylogeny is constructed based on the *n* top-scoring strain taxa (default: *n* = 30). First, the strain genome fasta file is filtered to include only sequences of these top-scoring strain taxa. Subsequently, MAFFT^58^ is executed in auto mode to perform a multiple sequence alignment (MSA). The resulting MSA serves as input for IQ-TREE,^59^ which it uses to reconstruct a phylogenetic tree at the strain level. Following the phylogenetic reconstruction, the nodes of the tree, initially named after the GenBank accessions of the corresponding genomes, are renamed to match the corresponding taxon IDs. This renaming step is important for downstream tools, such as PepGM,^29^ thus ensuring consistency and enabling further analyses.

### Top-Scoring DB Search

Following the construction of the phylogeny based on the top-scoring strain taxa, an optional final search step is performed. This step exclusively uses the proteomes of these top-scoring strain taxa. Similar to previous searches, this step results in a list of the top *n* candidate strains, each accompanied by their respective weighted PSM scores. This approach enhances results by reducing database size and minimizing duplicates. Consequently, the database contains fewer entries, potentially yielding more PSMs for taxa present in this refined proteogenomic database and offering more reliable results.

### Additional Possibility to Compute the Database Suitability

To assess the quality of input databases, MultiStageSearch offers an adapted version of the protein sequence database suitability method introduced by Johnson et al.^60^ In this approach, the software Novor^61^ is used to generate *de novo* peptide sequences from the MS/MS spectra contained in the provided MGF file. These *de novo* peptides are labeled with a unique header prefix “*NOVOR*_*PEPTIDE*_”, and then merged with the database being used in the current search step. Subsequently, a standard database search is applied to this augmented database. Using the resulting report from PeptideShaker or MS2Rescore, MultiStageSearch computes the proportion of “real” database hits compared to Novor-generated peptide hits.

While Johnson et al.^60^ perform a reranking of PSMs with nearly identical scores, this step is omitted in MultiStageSearch because our workflow does not rely on ranking PSMs. Instead, when identified peptides with the same theoretical mass can be assigned to both “real” database hits and Novor peptide hits, only the “real” database hits are retained, while the Novor peptide hits are filtered out. After filtering, the suitability of the database is assessed in a manner similar to Johnson et al. by:

3 $$suitability_{D}=\frac{\sum PSM_{D}}{\sum PSM_{D}+\sum PSM_{N}}$$

In eq 3 PSM_D_ refers to a PSM identified from the database, while PSM_N_ corresponds to a PSM found in the *de novo* peptide sequences. Thus, the database suitability is calculated as the proportion of the hits from the “real” database relative to the total number of identified hits. This calculation is performed at each search step to evaluate the appropriateness of the databases used. The resulting database suitability value helps the user determine whether the reference databases are appropriate. On a side note, a low suitability value may also indicate poor sample quality, as low-quality samples lead to poor spectral quality and, consequently, fewer identifications. However, it is important to note that the calculation cannot distinguish between poor database suitability and poor sample quality when only one database is used.^60^

By calculating the database suitability across multiple search steps, each using different reference databases, users can gain insights: consistently low suitability across all steps indicates poor sample quality, while low suitability in a single search step suggests an improperly composed reference database. In general, a high database suitability value indicates more trustworthy results from the workflow compared to a low suitability value.

### Results Output and Visualization

MultiStageSearch computes four different metrics for the inference of the strains. Among those are the weighting described in the section “Reference Database Search” and the amount of identified PSMs per strain. Finally, to visualize the results of the database searches, the similarity comparisons and the suitability of the databases, several plots are created. These show the different results: the weighting of taxa for the different search steps, the similarities of proteomes and identified peptidomes, and other metrics. The similarity of the identified peptidomes is calculated similarly to the pairwise similarity of the proteomes *S*_*P*1,*P*2_, except only identified ORFs are considered.

Additionally to the plots, MultiStageSearch creates output that is readable by potential further downstream analysis pipelines such as PepGM.^29^ This includes:

- a phylogenetic tree in newick format,
- a CSV file with accessions and taxon IDs for identified strains,
- a CSV file containing final scores (weights) for top-scoring strains,
- a CSV file containing the identified peptides, the sequences of the peptides, the score of the PSMs, the taxon IDs of the strain and the ancestor.

To provide a comprehensive overview of the results to the user, both plots and essential log files are compiled into an interactive HTML report. This report is generated using R-Markdown,^62^ enabling easy navigation and understanding of the most critical information derived from the MultiStageSearch analysis.

### Benchmark Samples

To evaluate the performance of MultiStageSearch against other tools like TaxIt^40^ and PepGM, several viral samples were tested. First, we applied MultiStageSearch to several purified virion samples used in previous studies to benchmark tools for viral identification at strain level. We use purified virions as a ground truth for benchmarking, because the exact viral strain and taxonomic content of the sample is known. This is not always the case for clinical samples, who additionally may have a complex background of host cells and other microbiome components depending on sample collection methods. These are two Cowpox samples (PXD003013, PXD014913) of which both are the strain “Brighton Red”, a Hendra virus sample (PXD001165, strain “HeV/Australia/1994/Horse18”), an avian bronchitis sample (PXD002936, strain “Beaudette CK”), a Adenovirus sample (PXD004095, strain/serotype “2”), a Herpes simplex 1 sample (PXD005104, strain “F”) and two SARS-CoV-2 samples (PXD018594, PXD025130) both of lineage B. Additionally, we included clinical samples with a more complex background: these are stool samples from children with confirmed gastrointestinal viral infections. The disadvantage of these clinical samples is that the exact viral strain is unknown, but they still complement the benchmark samples effectively. Two negative controls corresponding to supernatant of cell cultures used for virus culturing are also included. The samples are available under the PRIDE identifier PXDPXD036663. To further showcase the applicability of MultiStageSearch on bacteria, a sample containing Pseudomonas aeruginosa (PXD026634) is included. The strain of this sample is “CCUG 51971”. Notably, this strain is not present in the NCBI Taxonomy and other tools dependent on the NCBI Taxonomy are therefore not able to infer the correct strain. We chose this bacterial strain specifically to showcase the use of MultiStageSearch for bacterial strains not represented in the NCBI taxonomy. For standard bacterial strains, identification methods for proteome samples already exist.^26^ All sample data sets are publically available in the PRIDE^63^ database.

## Results

### Exploration of the Database Bias

As previously hypothesized by the authors of PepGM,^29^ the bias of the NCBI protein database toward certain pathogens can impede accurate identification. This bias is particularly evident in the herpesvirus data within the NCBI protein database. In Figure 2a, the distribution of protein sequences corresponding to the taxon IDs of strains present in the NCBI taxonomy are visualized. Notably, strain 17 is overrepresented, while only a limited number of other strains can be found, which impacts the performance of methods relying on the NCBI taxonomy for strain-level identification.

**Figure 2:**
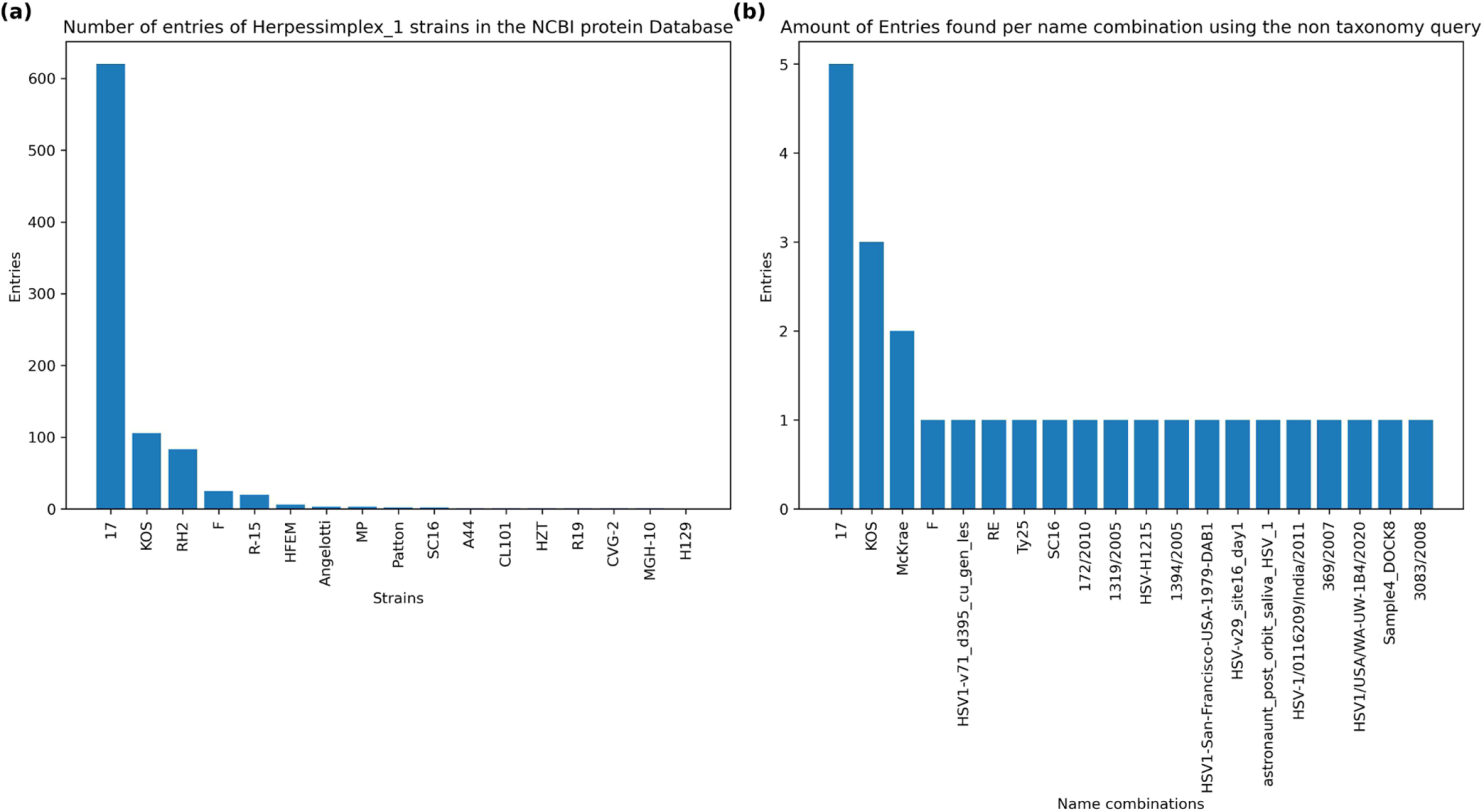
(a) Overrepresentation bias of the NCBI protein database compared to the overrepresentation bias of the result from the (b) MultiStageSearch query for the human herpesvirus 1. Only the first 20 name combinations are shown.

In contrast, the MultiStageSearch query, using the name combinations as described in section “Download and Processing of Genomic Data” effectively reduces this bias, as demonstrated in Figure 2b. By optimizing the query strategy, MultiStageSearch is able to provide a more balanced representation of strains, mitigating the overrepresentation issue observed in the standard NCBI taxonomy.

### Overall Performance Comparison against State-of-the-Art Methods

To demonstrate the taxonomic assignment capabilities of MultiStageSearch, we conducted a benchmark against PepGM and TaxIt using the selected nine samples described in the “Benchmark Samples” section. The benchmark results for PepGM and TaxIt were obtained from the original study of PepGM.^29^ Additionally, we now analyzed four stool samples with a more complex background and two negative controls as described in the methods. We did not analyze them with TaxIt or PepGM as these tools are currently not adapted to analyze samples with more complex backgrounds. While MultiStageSearch successfully identified the correct strains for most samples, as shown in Table 1, TaxIt was not benchmarked on the SARS-CoV-2 samples. Both PepGM and MultiStageSearch accurately identified the correct species, whereas the bacterial sample was not tested with PepGM and TaxIt due to their reliance on the NCBI Taxonomy, which limits their ability to infer strain-level information.

**Table 1:**
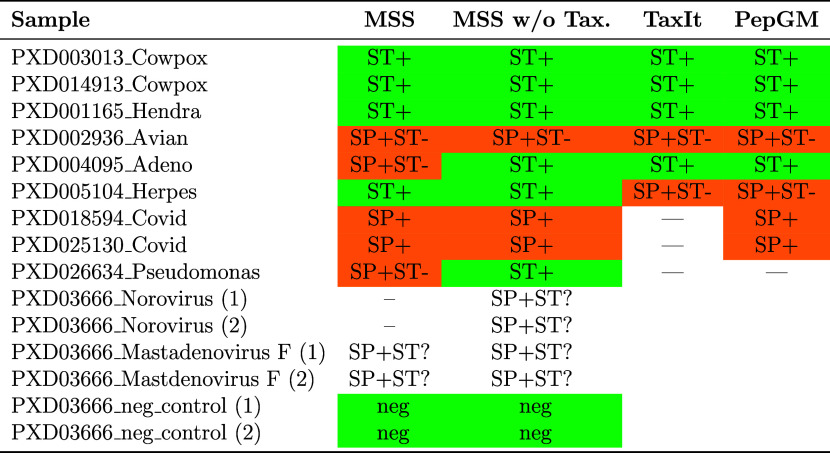
Overview of the Taxonomic Identification Performance of Different Methods^a^

a ST+: Correct strain identification. SP+: Correct species identification. --: Not applicable or not tested. ST?: exact strain unknown.

As shown in Table 1, the strain of the avian bronchitis sample could not be identified by any of the methods. However, the overall results demonstrate that MultiStageSearch, which is not dependent on the NCBI Taxonomy, consistently outperforms TaxIt and PepGM. Notably, MultiStageSearch correctly identified both the strains of the Herpesvirus and P. aeruginosa samples, whereas TaxIt and PepGM did not. The results indicate that MultiStageSearch is able to make use of the advantages of genomic data as well as the independence of the NCBI taxonomy to achieve higher strain-level identification accuracy for most of the samples tested. The results are discussed in more detail in the following.

### Taxonomic Identification Accuracy Using the NCBI Taxonomy

When using the NCBI Taxonomy for querying genomic strain-level data in the NCBI nucleotide database, MultiStageSearch accurately identified the correct strains for the two Cowpox virus samples, the Hendra virus sample as well as the Herpes virus sample as shown in Table 1. In Figure 3, the final results for the Herpes virus sample are shown. By using the weights computed by eq 1 and summed as described in Section Reference DB *Search*, MultiStageSearch identified the correct strain “F”, demonstrating its effectiveness in overcoming database bias. In contrast, PepGM incorrectly identified the herpesvirus strain 17 for this sample, and the original authors attributed this misidentification to database bias.^29^ In fact, the herpesvirus 1 strain 17 is distributed by a scientific retailer.^64^ Because it is readily available, it is more extensively used in experiments, and corresponding protein sequence are uploaded to online databases in greater number: strain 17 has 670 protein entries in the NCBI database to date (as of May 2024), but strain F has only 25.

**Figure 3:**
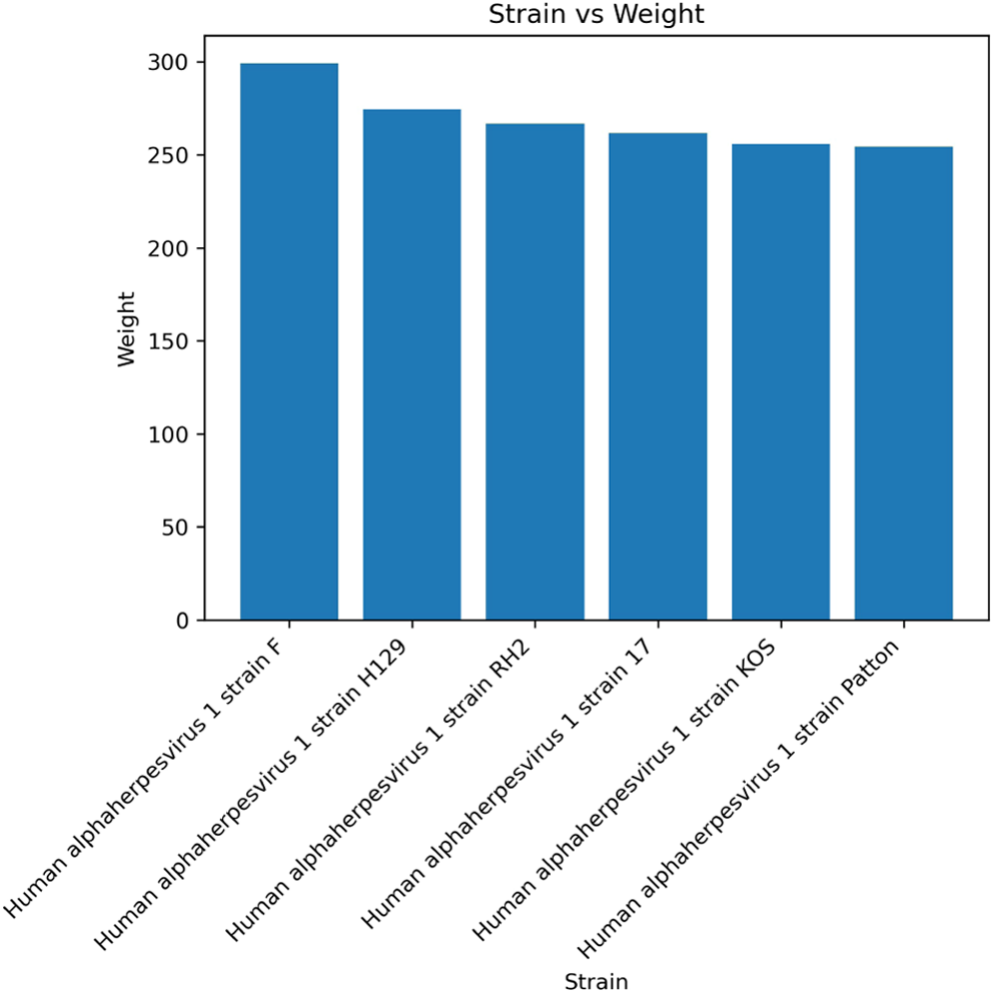
Bar plot showing the final weights of the top-scoring database search for the herpes sample.

The SARS-CoV-2 samples, however, could not be identified at the subspecies level using the NCBI taxonomy. This is due to the missing data for SARS-CoV-2 in the taxonomy. Although taxonomic data was available for the avian bronchitis and adenovirus samples, both were incorrectly identified. As shown in Figure 4, the two strains “Beaudette CK” and “Beaudette” have the same weight for avian bronchitis, making differentiation difficult. Figure 5 illustrates the pairwise peptidome similarity between the proteogenomic sequences for the avian bronchitis virus sample, revealing that the strains “Beaudette CK” and “Beaudette” share 100% similarity in their identified peptidomes. Therefore, MultiStageSearch, along with TaxIt and PepGM, failed to correctly identify the strain “Beaudette CK”. Peptidome heatmaps for the strains of all samples analyzed are shown in the Supporting Information in Figures S3–S8. These are automatically computed by the MultiStageSearch workflow. They give an indication of how similar the peptidomes—i.e., the sets of detected peptides—are across different strains. The more similar a peptidome, the more difficult it becomes to confidently distinguish between strains. We discuss this in more detail for the avian bronchitis virus sample (Figure 5). The sequence similarity between candidate strains is also shown in the bar blot of peptide counts per strain: if two strains have a very similar peptidome, their number of identified peptides will be very close.

**Figure 4:**
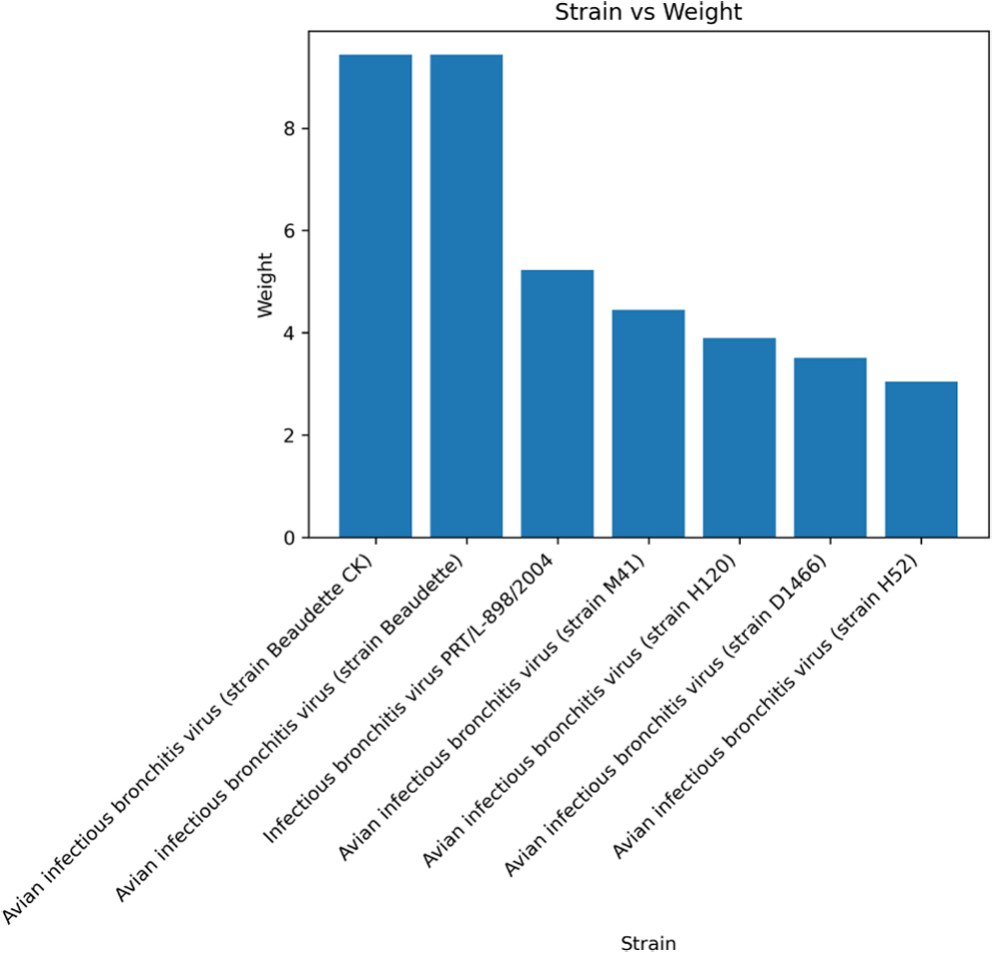
Bar plot showing the final weights of the top-scoring database search for the avian bronchitis sample.

**Figure 5:**
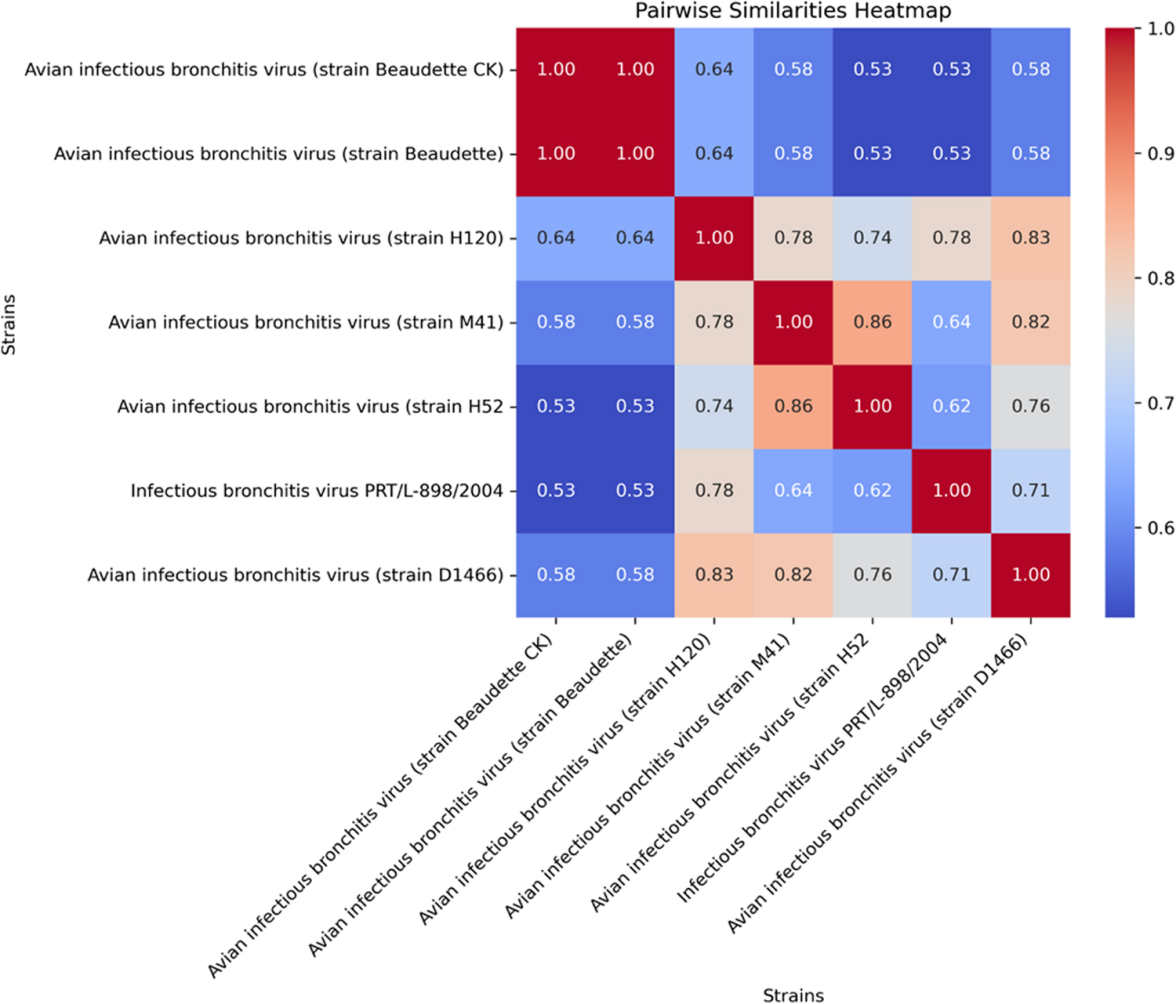
Heatmap of the similarities of identified peptidomes of the top-scoring genomes for the avian bronchitis sample.

The situation is different for the adenovirus. In this case, the reference database search reported two species with the highest weights, as shown in Figure 6. These species correspond to “Human mastadenovirus C” and “Human adenovirus 2”. It is important to note that in the NCBI taxonomy, “Human adenovirus 2” is a descendant of “Human mastadenovirus C” without a specified rank. When querying the database using the name combinations as described in section “Download and Processing of Genomic Data”, no strain-level genomes were found. This is because the query searches for the same names for both species and strain. An example of this query is Human adenovirus 2 AND ((strain Human adenovirus 2C­[All Fields]) tor (Human adenovirus 2C­[Title])) AND “complete ”[Title].

**Figure 6:**
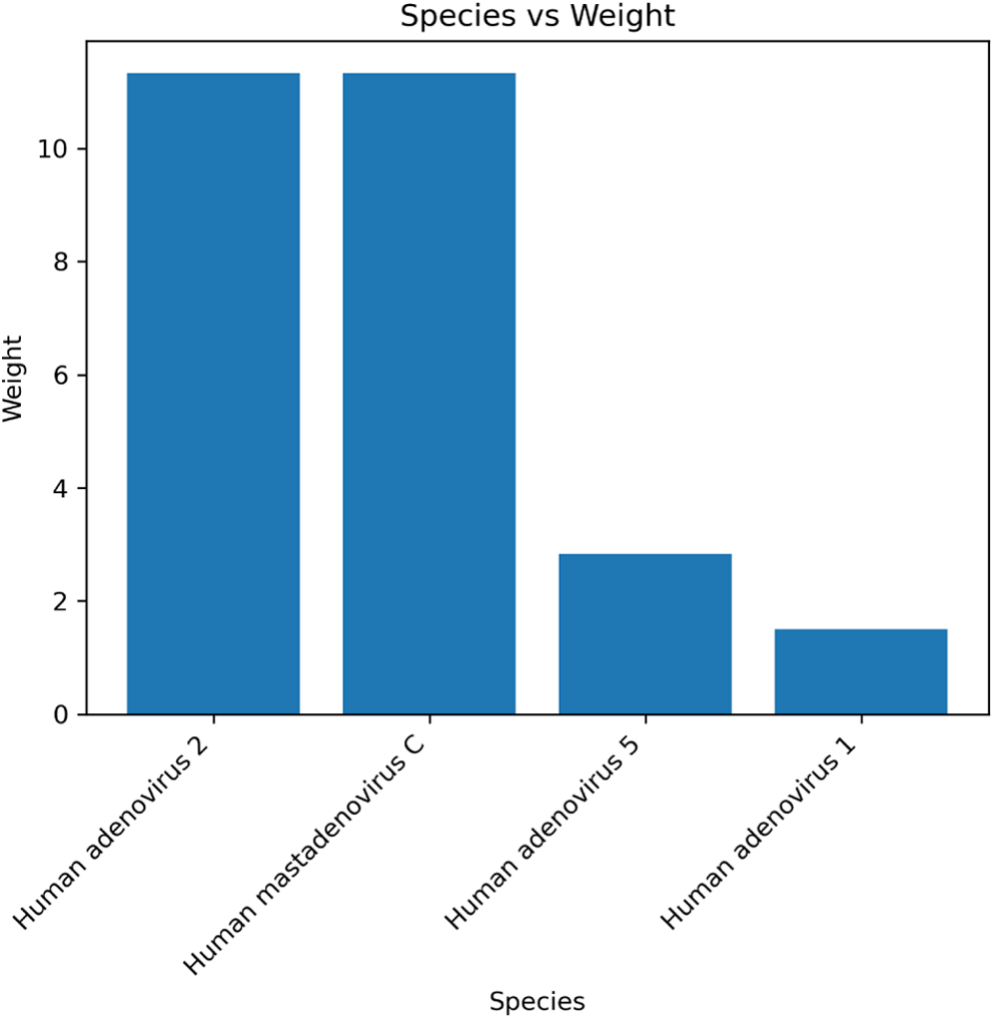
Barplot showing the identification results as species level against the reference database for the adenovirus 2 sample.

This query strategy, therefore, did not find the correct strain-level genome for the adenovirus sample.

### Improved Strain-Level Performance without NCBI Taxonomy Use

The results in Table 1 show that MultiStageSearch identified the correct strain with even higher accuracy than TaxIt and PepGM when it does not rely on the NCBI taxonomy. Instead of using the often nonexistent strain-level NCBI taxonomy, MultiStageSearch searches for entries linked to the taxon ID of the species. A significantly higher number of strains and isolates were found in the NCBI database using this approach and the strains were still identified correctly for most samples. However, for SARS-CoV-2, the NCBI taxonomy at levels lower than species was not defined at the time of writing. Despite this, millions of proteins and nucleotide sequences for SARS-CoV-2 are available in the NCBI database. The standard query of MultiStageSearch, designed to search for name combinations based on the NCBI taxonomy, is unable to process this vast amount of data within a reasonable time frame. Consequently, the correct strain cannot be found for SARS-CoV-2.

The other virus sample that could not be correctly identified at the strain level was the avian bronchitis sample. In this case, a similar issue as with the approach using the NCBI taxonomy arises. Figure 7 shows two key findings: (1) MultiStageSearch found only very few PSMs for the top-scoring genomes with only up to 36 PSMs per strain/isolate, likely due to the quality of the sample itself. (2) The three top-scoring strains had the exact same amount of PSMs. Similar to the issue with the NCBI taxonomy approach, the identified peptidomes of the top-scoring genomes are identical. Therefore, they cannot be distinguished. Furthermore, the filtering of duplicate proteomes did not work here, since the strain names were too different.

**Figure 7:**
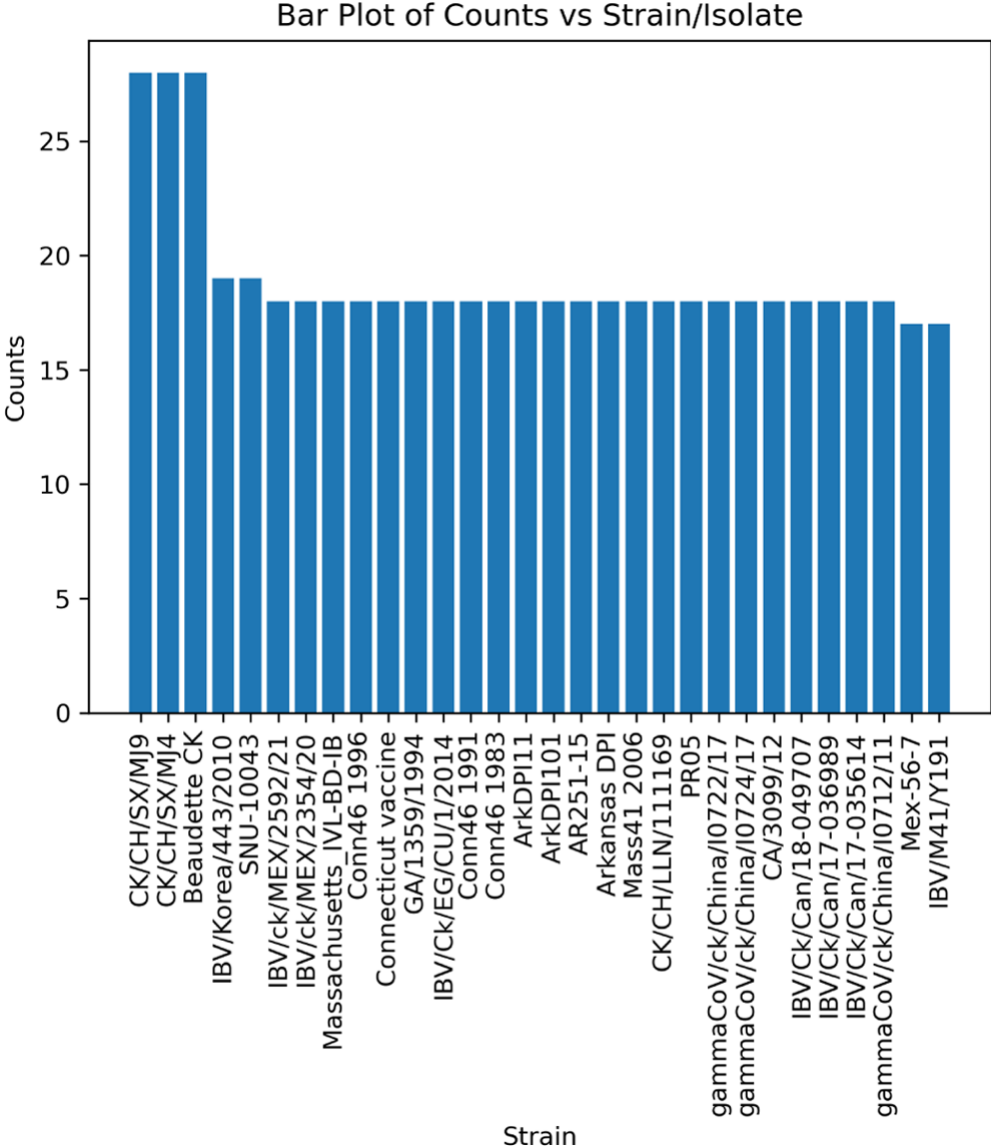
Bar plot showing the number of PSMs per strain/isolate of avian bronchitis.

Table 2 shows the advantages the independence of the NCBI taxonomy and the proteogenomic approach. It gives the number of strain references found in the NCBI taxonomy for the purified virion species we used for benchmarking. While most viruses do not have many strain references in the NCBI taxonomy,^31^ many more genomes are found that can be assigned to different strains and isolates not represented in the taxonomy. Therefore, the genetic variance of most species is covered to a greater degree and the automatically created proteogenomic database covers more variance for a given species. By using an approach independent of the NCBI taxonomy, MultiStageSearch is able to use this increased variants coverage for better strain-level identification. The Cowpox virus has five strains in the NCBI taxonomy and 98 complete genomes were found using MultiStageSearch. Although there is only one strain (“Hendra virus horse/Australia/Hendra/1994”) present in the NCBI taxonomy for the Hendra virus, MultiStageSearch was able to find 19 complete genomes. For the avian bronchitis, adenovirus, and herpesvirus samples, MultiStageSearch found hundreds of complete genomes. However, for SARS-CoV-2, MultiStageSearch identified millions of complete genomes, showcasing the need for advanced bioinformatic solutions beyond those presented in this work, to effectively use this vast amount of data.

**Table 2:** Available Strains Per Species in the NCBI Taxonomy and the Number of Genomes Found by MultiStageSearch (MSS) Independently of the NCBI Taxonomy^a^

species	strains in taxonomy	(complete) genomes found by MSS w/o tax.
Cowpox Virus	5	98
Hendra Virus	1	19
Avian Bronchitis Virus	34	500
Human Adenovirus 2	44	361
Human Alphaherpesvirus 1	17	164
SARS-CoV-2	0	5,364,560
P. aeruginosa	382	500
Human Mastadenovirus F	7	154
Norovirus GII	14394	46,693

a Each genome can potentially represent a strain that is not represented in the NCBI taxonomy.

Figure 8 shows the results of MultiStageSearch without using the NCBI Taxonomy for the P. aeruginosa sample. The results are presented based on the number of identified PSMs, as this approach tends to be more robust using MS2Rescore compared to using weightings. It is visible that the strain “CCUG 51971” could be inferred with the highest amount of identified PSMs.

**Figure 8:**
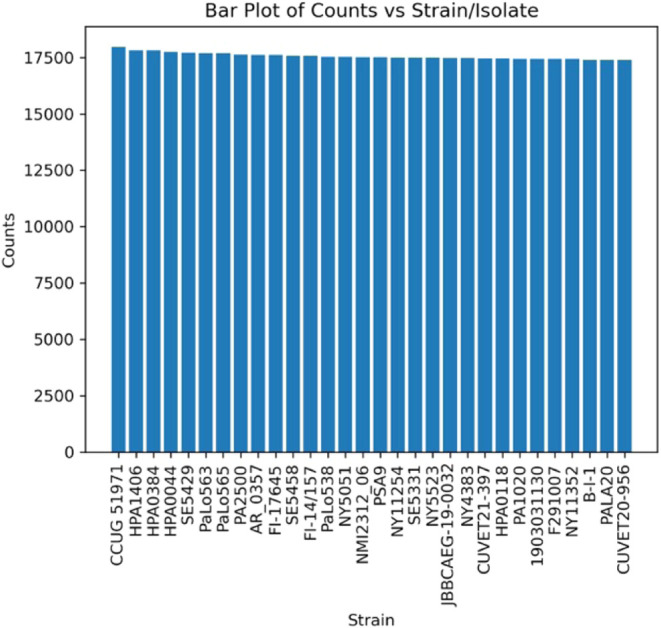
Bar plot showing the number of PSMs per strain/isolate of P. aeruginosa.

### Clinical Samples with Complex Backgrounds

To assess the performance of MultiStageSearch on samples with more complex backgrounds, we included four samples from children with confirmed viral infections of the gastrointestinal tract. The drawback here is that for these samples, the exact viral strain is not known, therefore we can only draw limited conclusions concerning the correctness of the identification. Interestingly, for all four samples, MultiStageSearch already identified the correct viral species in the first, general search against RefSeq Viral. This is likely attributable to the fact that both species are well-characterized human pathogens with extensive representation in viral reference sequence databases. An example of the first search results is shown for the Norovirus(2) sample in Figure 9. The clade (this is a taxonomic level below species) identified is “Norovirus GII”, which is likely correct as this is the most common pathogenic human norovirus clade.^65^ The same observation was made for the Norovirus(1) sample, where the first search immediately identified the clade “G.II.17”, also increasingly identified in Europe according to the ECDC.^66^

**Figure 9:**
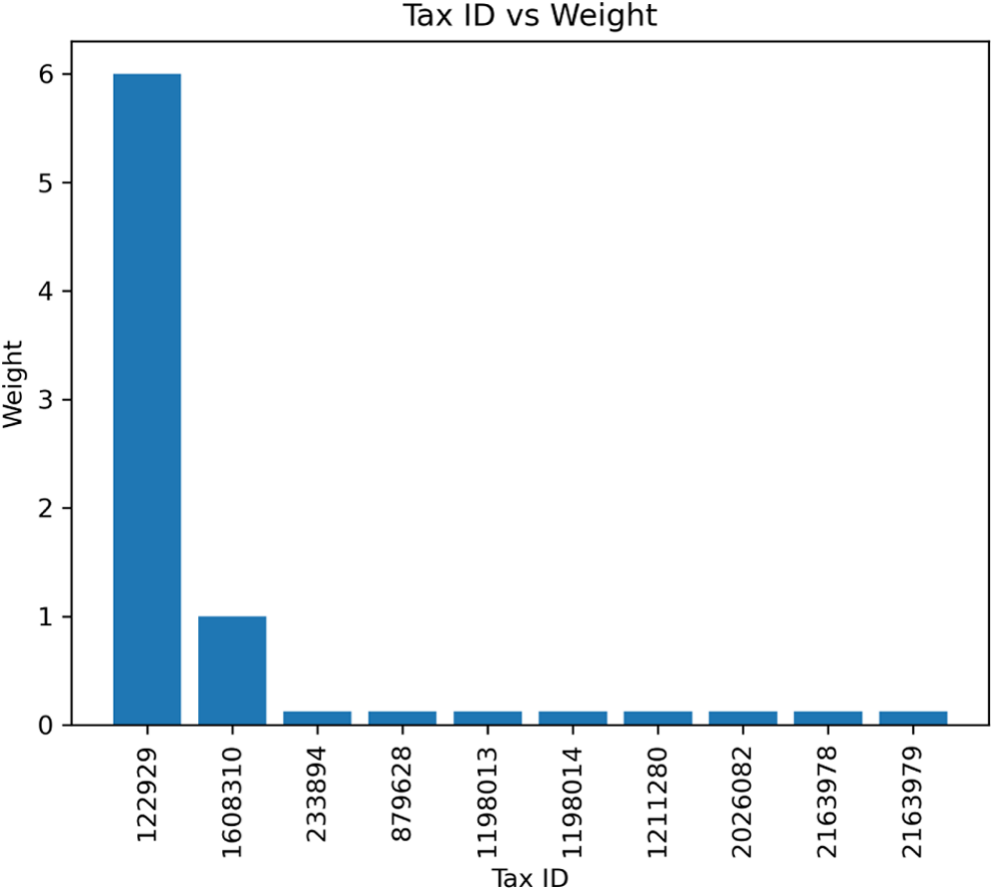
Bar plot showing the number of PSMs per species for the Norovirus(2) sample. The first taxid 122929 is “Norovirus GII”, the second one, 1608310, is a phage.

In the second, strain-level search, MultiStageSearch identified several isolates of the genotype “GII.4” and remained at the “GII.17” clade for each Norovirus sample. The number of PSMs is only 5 in the first case and 6 in the second. In fact, the reference search database at strain level is very large and contains many strongly overlapping genomes, as the norovirus is a very common pathogen that is frequently sequenced. It is also extensively represented in the NCBI Taxonomy (see Table 2). In contrast to Sars-CoV-2, it is a well-characterized pathogen that has been studied for many years. This also explains why the Norovirus samples did not yield any identifications when running MultiStageSearch in the NCBI Taxonomy-dependent mode: there are over 14,000 isolates referenced in the taxonomy.

The Mastadenovirus samples analyzed were also identified with the correct species “Human Mastadenovirus F” in the first search. The strain-level search in NCBI-taxonomy dependent mode identified the serotype “Human mastadenovirus 40” for both samples, but this was the only strain referenced in NCBI and therefore does not represent a reliable result. The NCBI Taxonomy-independent search identified multiple Human mastadenovirus F associated genome references, but the detected peptidomes mapped to these strains were too similar to allow for a conclusive strain-level identification. The heatmap depicting this is shown in the Supporting Information in Figure S8.

### Assessing the Impact of the Search Space Using the Database Suitability

The choice of database strongly influences identification outcomes. While the user provides databases for host filtering and reference database searches, MultiStageSearch dynamically creates the proteogenomic database based on intermediate results. Figure 10 shows the database suitabilities for the four search steps using the query approach without relying on the NCBI taxonomy, focusing on the Cowpox sample (PXD003013). Suitabilities were computed following the procedure described in the methods section above.

**Figure 10:**
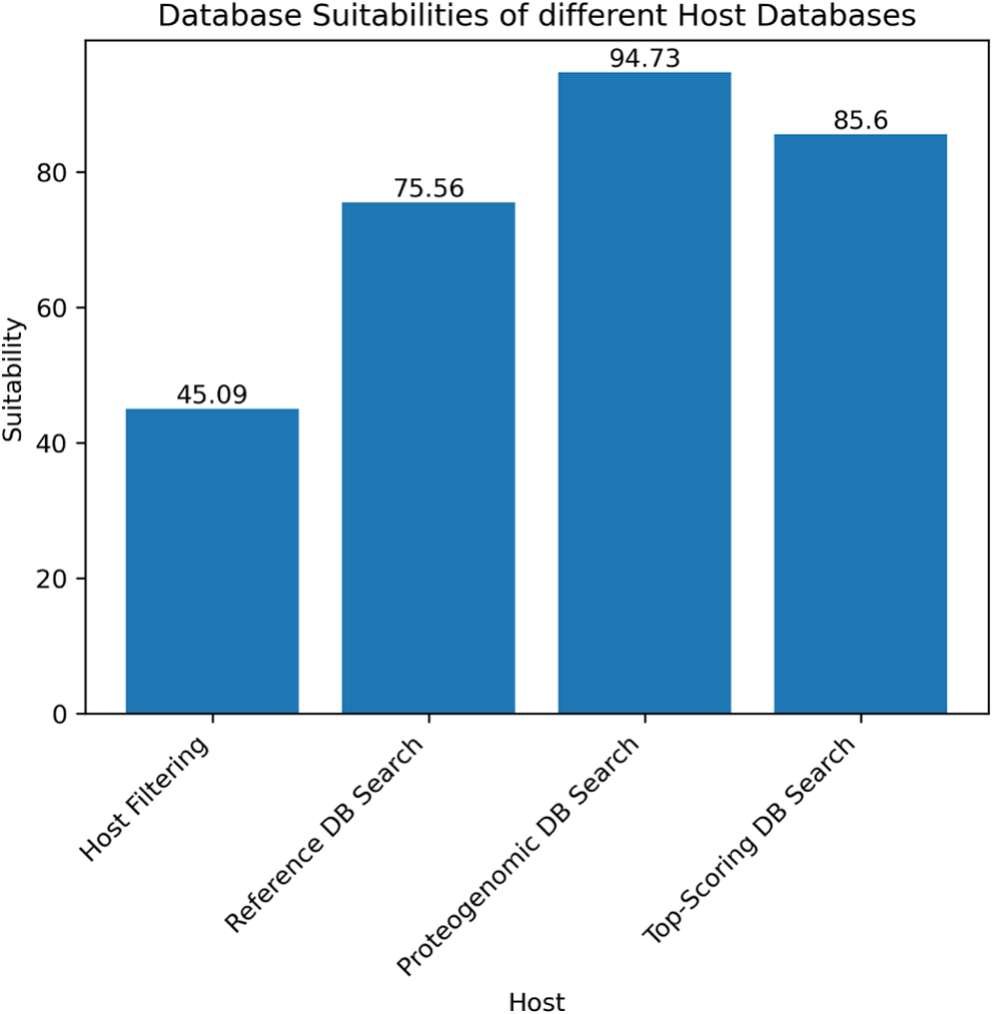
Bar plot showing the database suitability rates of the PXD003013_Cowpox sample using the query approach without the taxonomy.

The user-provided databases, in this case the host (Rattus norvegicus) combined with the cRAP database and the RefSeq viral database, demonstrate suitability rates of 45.09 and 75.56% respectively. Notably, the automatically generated proteogenomic database exhibits the highest suitability at 94.73%. This emphasizes the benefit of generating a proteogenomic database tailored to the specific search context, making more accurate and comprehensive identification possible.

Figure 11 shows the database suitability rates for the herpes sample, revealing significant differences. While the suitability of the host database is higher compared to the one for the Cowpox sample (PXD003013) in Figure 10, the suitability of the reference database is much lower. Several factors could contribute to this disparity: (1) The database may not include the correct species of the sample. (2) The sample could contain more spectra that are not from the species analyzed. (3) The overall quality of the sample may be low. All these factors collectively contribute to lower database suitability by reducing the number of database peptides found and increasing the number of *de novo* peptides.

**Figure 11:**
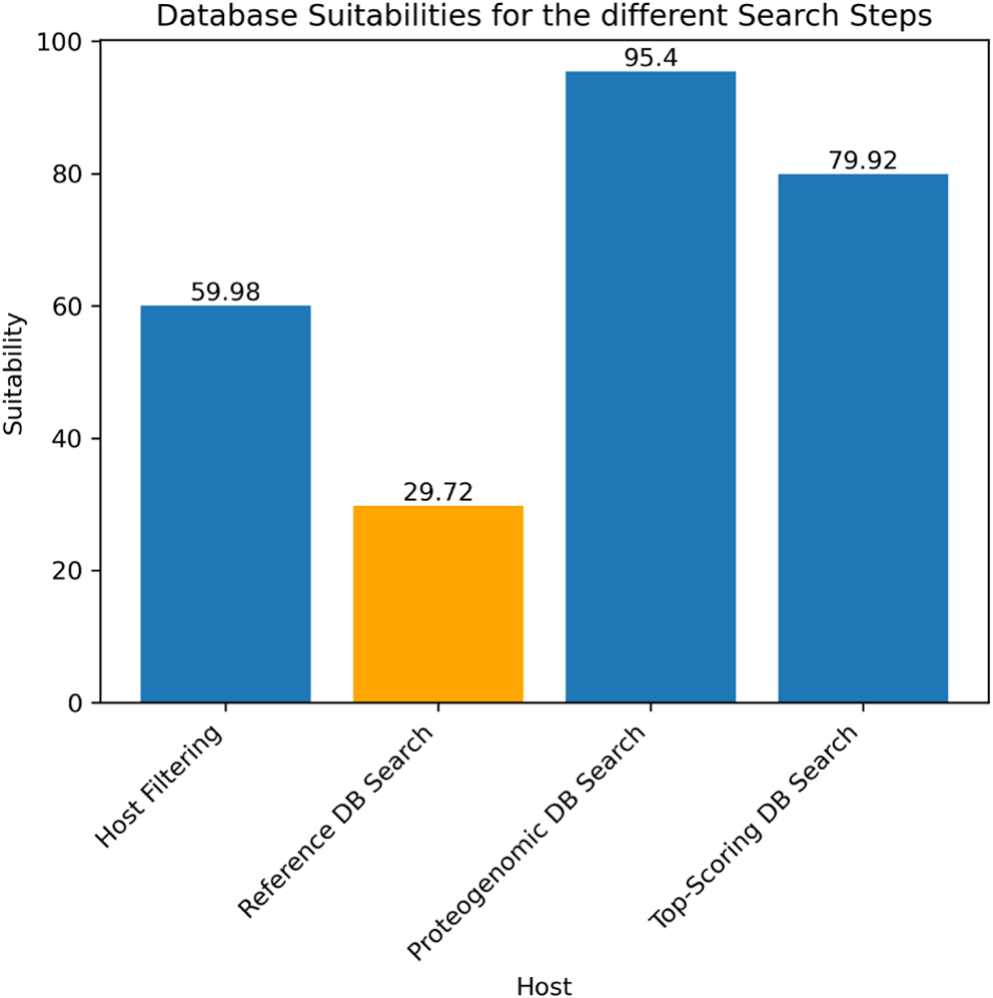
Bar plot showing the database suitabilities of the herpes sample using the query approach without the taxonomy.

However, the high suitability of over 95% in the proteogenomic search demonstrates that this approach can effectively create a database tailored to the sample, even in cases where the reference database suitability or sample quality is not particularly high.

Figure 12 displays the database suitabilities for the Hendra virus sample. Each suitability value, except for the host database (44.49%), is remarkably low, ranging from 5.53 to 6.68%. This suggests that the sample contains very little of the virus itself, as indicated by the significantly higher suitability of the host database compared to databases containing viral references. Nevertheless, MultiStageSearch successfully identified the correct strain “HeV/Australia/1994/Horse18”, which might partly be due to the relatively small number of strains available as references additionally to the correct reference strain, with only 19 genomes compared to 98 for the next lowest number (Cowpox virus, see Table 2).

**Figure 12:**
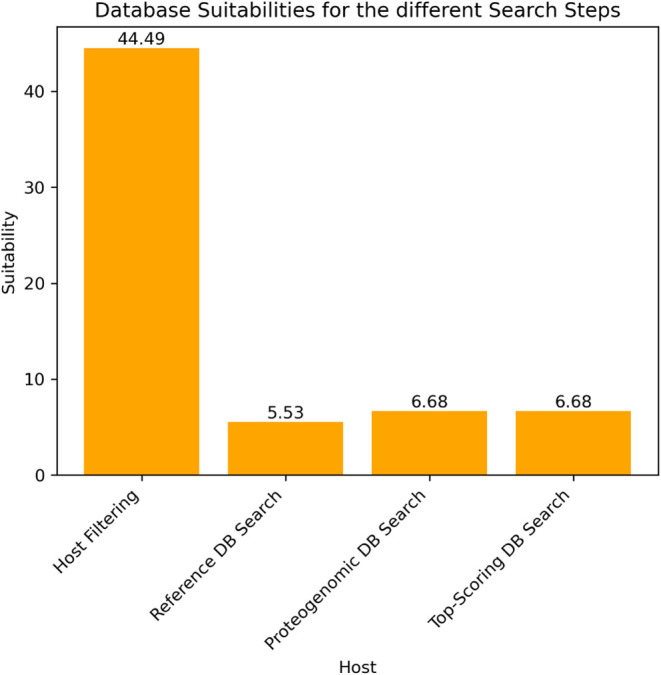
Bar plot showing the database suitabilities of the Hendra virus sample using the query approach without the taxonomy.

To determine a threshold for database suitability, below which the user is advised to investigate the suitability of the database and the quality of the sample, the herpes sample was tested with four other animal host databases (R. norvegicus, Pteropus alecto, Gallus gallus, and Chlorocebus sabaeus). The database suitability rates dropped from 59.98 to 50.84%, 41.25, 42.34, and 51.08%, respectively, as illustrated in Figure S2 in the Supporting Information. Therefore, it is recommended to investigate both the sample quality and the database composition when the suitability rate falls below 45%.

## Discussion

The increasing demand for precise and personalized therapeutic interventions necessitates the development of fully untargeted diagnostic techniques. We have developed an multistep proteogenomic workflow designed for strain-level identification of viral and bacterial samples. This workflow addresses the challenges of both sensitivity and database bias limitations in current MS/MS-based taxonomic identification methods.

The results of this study show that MultiStageSearch consistently identifies mainly the correct strain in viral and bacterial proteomic samples, thereby operating independently of the NCBI taxonomy. The integration of genome data for strain-level searches contributes to more reliable identifications, given that vast amounts of sequenced genomes are available.^8^

Unlike traditional methods that rely solely on proteome databases, MultiStageSearch integrates genomic data with proteomic data, thus offering a more balanced and comprehensive approach to taxonomic analysis proteogenomics increases the accuracy of identifying pathogens. This is particularly useful for detecting novel or mutated strains that might be missed by proteomics alone. In addition, our approach of generating a tailored proteogenomic reference database by filtering duplicate proteomes and clustering identical ORFs mitigates the over-representation of model strains in reference databases, thus enhancing the accuracy of taxonomic identification at the strain level. For instance, our method successfully identified strain “17” for the herpesvirus, while the conventional proteomics-based methods were unsuccessful. Our dual strategy addresses the biases inherent in proteome databases and provides a robust framework for taxonomic identification.

The use of the NCBI nucleotide database via the NCBI taxonomy posed challenges related to the annotation of the taxonomy itself. For example, with the adenovirus sample, the query failed to identify “Adenovirus 2” due to its undefined taxonomic rank, resulting in ineffective name combinations that did not yield the correct strain. This highlights a significant limitation: the lack of strain-level taxonomy identifiers in the nucleotide database, which hampers accurate strain identification.

MultiStageSearch provides a solution by querying the nucleotide database using only the species-level taxonomy identifiers and bypassing the NCBI taxonomy. This method can even identify strains not present in the existing taxonomy, thus circumventing the issues related to missing annotations.

Despite these advancements, certain species remain challenging. One example is the avian bronchitis sample. Similarly to PepGM, the detected peptidomes of the top-scoring taxa were identical, even when using our proteogenomic approach. One possible explanation is that the strains “Beaudette” and “Beaudette CK” are too similar to differentiate them using MS-based technology. Another possibility is that not enough viral proteins were present or sufficiently enriched in the sample. Corroborating this hypothesis, Figure 1 in the Supporting Information shows that the database suitability for the proteogenomic search for this sample was only 48.75%, compared to over 90% for most other samples in the benchmark.

SARS-CoV-2 represents another challenging case for strain-level identification. The rapid mutation rate during the recent pandemic resulted in millions of humans being infected.^67,68^ Scientists around the world analyzed the virus and uploaded millions of mainly uncurated genome and proteome sequences. The lack of subspecies or strain-level classification in the NCBI taxonomy further complicates accurate identification. This situation underscores the importance of developing methods capable of identifying viral strains that have not yet been cataloged in standard taxonomic references, particularly during epidemic outbreaks and rapid evolutionary changes.

Given the millions of SARS-CoV-2 genomes in the NCBI nucleotide database, standard query methods used for most viral species are insufficient for accurate lineage classification in this case. To address this challenge, we developed an extended module of MultiStageSearch, termed the COVID mode, which specifically targets subspecies resolution by making use of pango-lineages.^69^ The COVID mode’s functionality and implementation are detailed in the supplementary data. However, the method’s accuracy may be affected by the high redundancy of closely related sublineages and the aliasing system used to represent them. The norovirus samples, for which tens of thousands of references are available, is another example that would benefit from a more up-to-date and organized taxonomic reference other than NCBI. Nextstrain^70^ is a resource with the potential to address this and could be included in MultiStageSearch in the future.

While our workflow showed a better performance than conventional proteomic taxonomic identification tools, specific challenges remain. Although proteogenomics is a powerful approach, it comes with the issue of an increased computational complexity. In particular, the integration of genomic and proteomic data is resource-intense in general. Furthermore, six-frame translation significantly increases the size of the database and the computational load, making the analysis more complex and time-consuming.

Another important aspect of taxonomic identification is the estimation of statistical confidence. While our workflow does not natively compute confidence estimates, this is due to the complexity of the multistep strain identification process, where the hierarchical nature of strain-level identification introduces dependencies that make direct confidence propagation challenging. Nevertheless, confidence estimation can be addressed in a separate downstream analysis using dedicated statistical modeling approaches such as PepGM.^29^ The output of MultiStageSearch can be used as input for PepGM, effectively replacing its original database search step.

## Outlook

To improve the results even further, the usage of additional databases such as the GISAID^71^ database in the case of SARS-CoV-2 and Influenza could be integrated. These databases provide extensive information that can complement and enrich current analyses, potentially improving accuracy and comprehensiveness. Other databases like Uniprot^32^ or real-time viral evolution tracking efforts such as Nextstrain^70^ can reduce the dependency on the NCBI databases and could therefore make the results more robust. In general, a diversification of data sources can mitigate biases and gaps inherent in single-database approaches, broadening the scope and reliability of taxonomic analyses. Looking ahead, optimizing several operational steps, such as enhancing the efficiency of filtering duplicate proteomes, will be important. Advances in algorithmic refinement and computational capabilities can lead to more streamlined processes, improving identification and ensuring higher fidelity in strain-level taxonomic analyses using proteogenomics.

## Conclusions

While strain-level identification using proteomics remains difficult, MultiStageSearch benefits from the advantageous use of different approaches, like proteogenomics and the novel combination of database queries and filterings. Implemented as a flexible Snakemake workflow, MultiStageSearch allows for easy integration of additional steps, making it highly adaptable for future expansion to meet the individualized needs of researchers.

In summary, MultiStageSearch represents an impactful advancement in the field of proteogenomics, with the potential to enhance the accuracy of strain-level identification. It points to the combination of proteomic and genomic databases as a possibility to overcome their individual limitations. Its potential applications in both research and diagnostic settings are extensive. Future developments will focus on refining these methods further, by improving their efficiency and exploring their applicability to a broader range of organisms, thereby enhancing the robustness and utility of proteogenomic analysis.

## Supplementary Material



## Data Availability

All data sets used are available through the PRIDE repository https://www.ebi.ac.uk/pride/ under their respective identifiers mentioned throughout the manuscript. The RefSeqViral and RefSeqBacterial databases are available through NCBI https://www.ncbi.nlm.nih.gov/refseq/. All code is available at https://github.com/rki-mf2/MultiStageSearch.

## Notes

List of packages used for the MultiStageSearch workflow (Table S1); bar plot showing the database suitabilities of the avian bronchitis sample using the query approach without the taxonomy (Figure S1); bar plot showing the database suitabilities of the herpes sample using different host databases (Figure S2); heatmap showing pairwise similarities between higher scoring proteomes for the Cowpox Virus sample PXD003013 (Figure S3); heatmap showing pairwise similarities between higher scoring proteomes for the Cowpox Virus sample PXD014913 (Figure S4); heatmap showing pairwise similarities between higher scoring proteomes for the Hendra Virus sample (Figure S5); heatmap showing pairwise similarities between higher scoring proteomes for the Adenovirus 2 sample (Figure S6); heatmap showing pairwise similarities between higher scoring proteomes for the herpes simplex virus (Figure S7); heatmap showing pairwise similarities between higher scoring proteomes for the Mastadenovirus (Figure S8); flowchart showing the workflow for the COVID mode (Figure S9); results of MultiStageSearch using the COVID mode for the PXD025130 SARS-CoV-2 sample (Figure S10); results of MultiStageSearch using the COVID mode for the PXD018594 SARS-CoV-2 sample (Figure S11) (PDF)
