# Supplementary material for: MultiStageSearch: An Iterative Workflow for Unbiased Taxonomic Analysis of Pathogens Using Proteogenomics
Source: J Proteome Res. 2025 May 19;24(6):2643–56. doi: 10.1021/acs.jproteome.4c00901 (PMC12150323; doi:10.1021/acs.jproteome.4c00901)
Supplement: Supplementary file 1 [file pr4c00901_si_001.pdf]

MultiStageSearch: an iterative workflow for unbiased taxonomic analysis of pathogens using proteogenomics

Julian Pipart,[†,⊥] Tanja Holstein,[‡,¶,†,§,‖,⊥] Lennart Martens,[‡,¶,§,‖] and Thilo Muth[*,†]

†*Data Competence Center MF 2, Robert Koch Institute, Berlin, Germany*

‡*CompOmics, VIB Center for Medical Biotechnology, VIB, Ghent, Belgium*

¶*Department of Biomolecular Medicine, Faculty of Medicine and Health Sciences, Ghent University, Ghent, Belgium*

§ *BioOrganic Mass Spectrometry Laboratory (LSMBO), IPHC UMR 7178, University of Strasbourg, CNRS, Strasbourg, 67000, France*

‖*Infrastructure Nationale de Protéomique ProFI FR2048, Strasbourg, 67087, France*

⊥*These two authors contributed equally to this work*

E-mail: mutht@rki.de

Contents

Python packages used **S2**

Additional Figures **S3**

Database suitabilities S3

Peptidome similarities S4

Table S1: List of packages used for the MultiStageSearch workflow

Package	Versions	Reference
pandas	1.5.3	[1]
biopython	1.81	[2]
numpy	1.22.4	[3]
ete3	3.1.2	[4]
ncbi-datasets-cli	16.4.7	[5]
iqtree	2.2.6	[6]
openjdk	11.0.13	—
mafft	7.520	[7]
pip	20.3	[8]
xtandem	15.12	[9]
chardet	4.0.0	—
percolator	3.5	[10]
psm-utils	0.7.3	[11]
ms2rescore	3.0.1	[12]
matplotlib	3.7.0	[13]
seaborn	0.12.2	[14]
emboss	6.6.0	[15]
libiconv	1.17	—
r-base	4.3.2	[16]
r-readr	2.1.5	[17]
r-knitr	1.45	[18]
r-dplyr	1.1.4	[19]
r-DT	0.33	[20]

Additional Figures

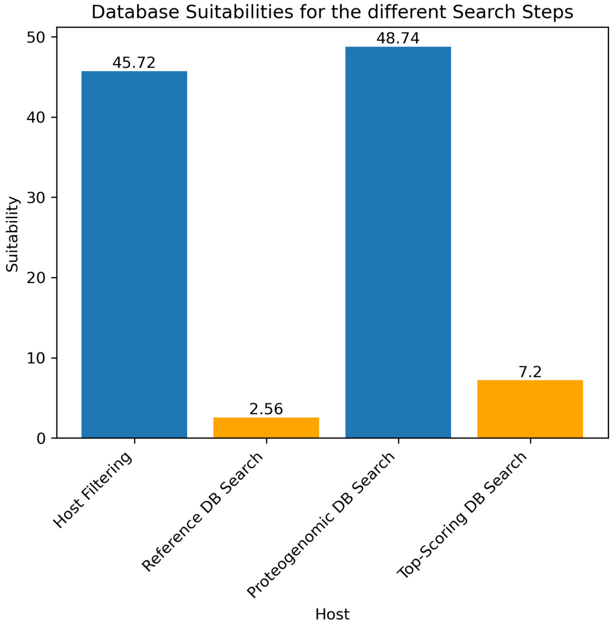

Figure S1: Bar plot showing the database suitabilities of the avian bronchitis sample using the query approach without the taxonomy.

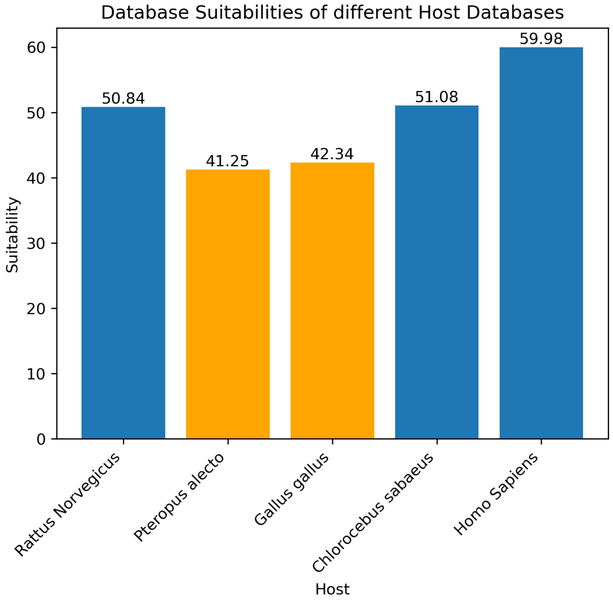

Figure S2: Bar plot showing the database suitabilities of the herpes sample using different host databases.

Peptidome Similarities

These plots shows additional peptidome similarity heatmap for the benchmark samples with known strain. The genome used are identified by their GenBank accession and ordered in decreasing number of peptide hits. All heatmaps are shown for the results of the MSS search independent of the NCBI taxonomy, as this includes a greater range of candidates genomes.

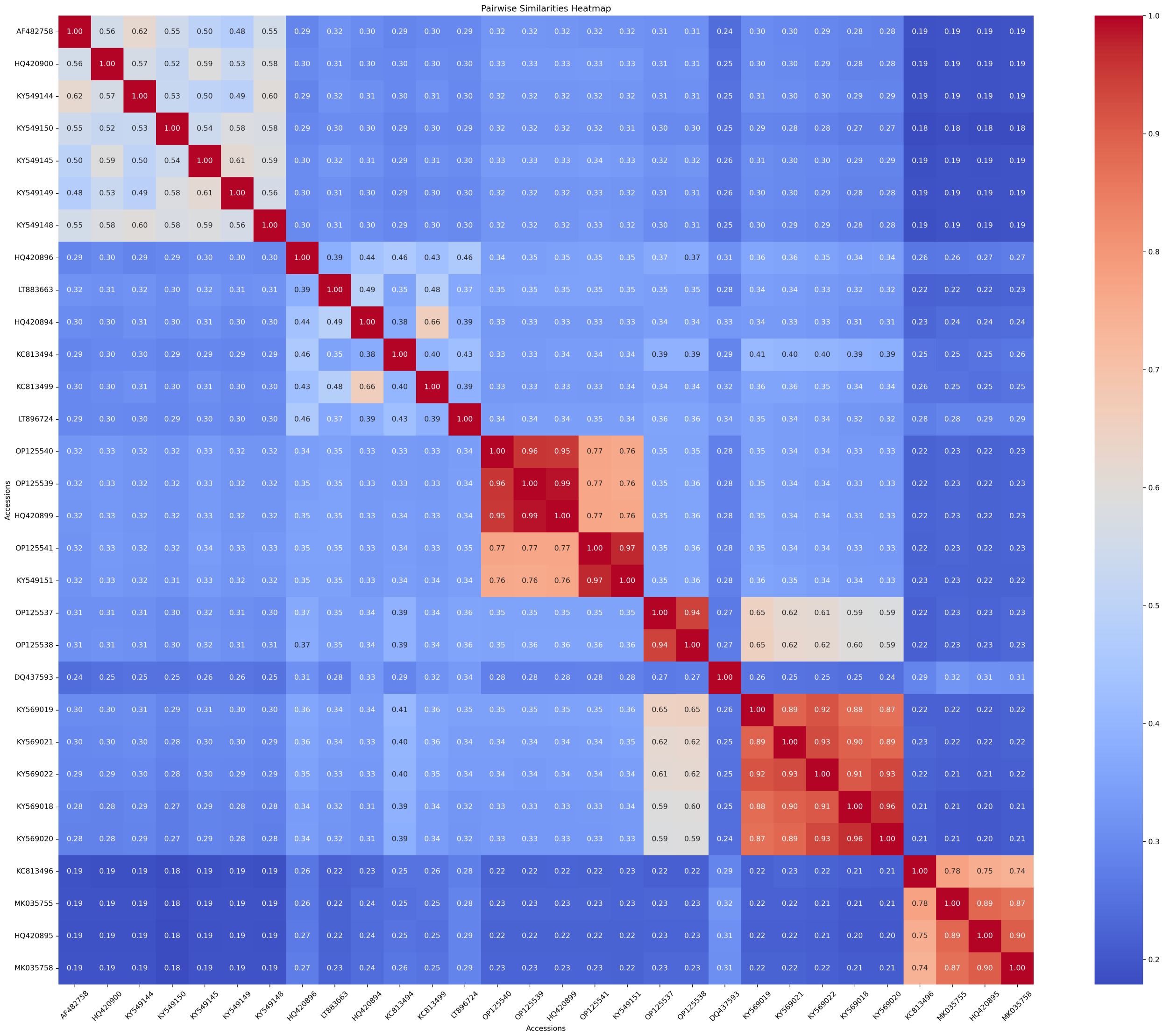

Figure S3: Bar plot showing the pairwise similarities between the higher scoring proteomes for the Cowpox Virus sample PXD003013. Similarities ranged between 0.1 and 1.

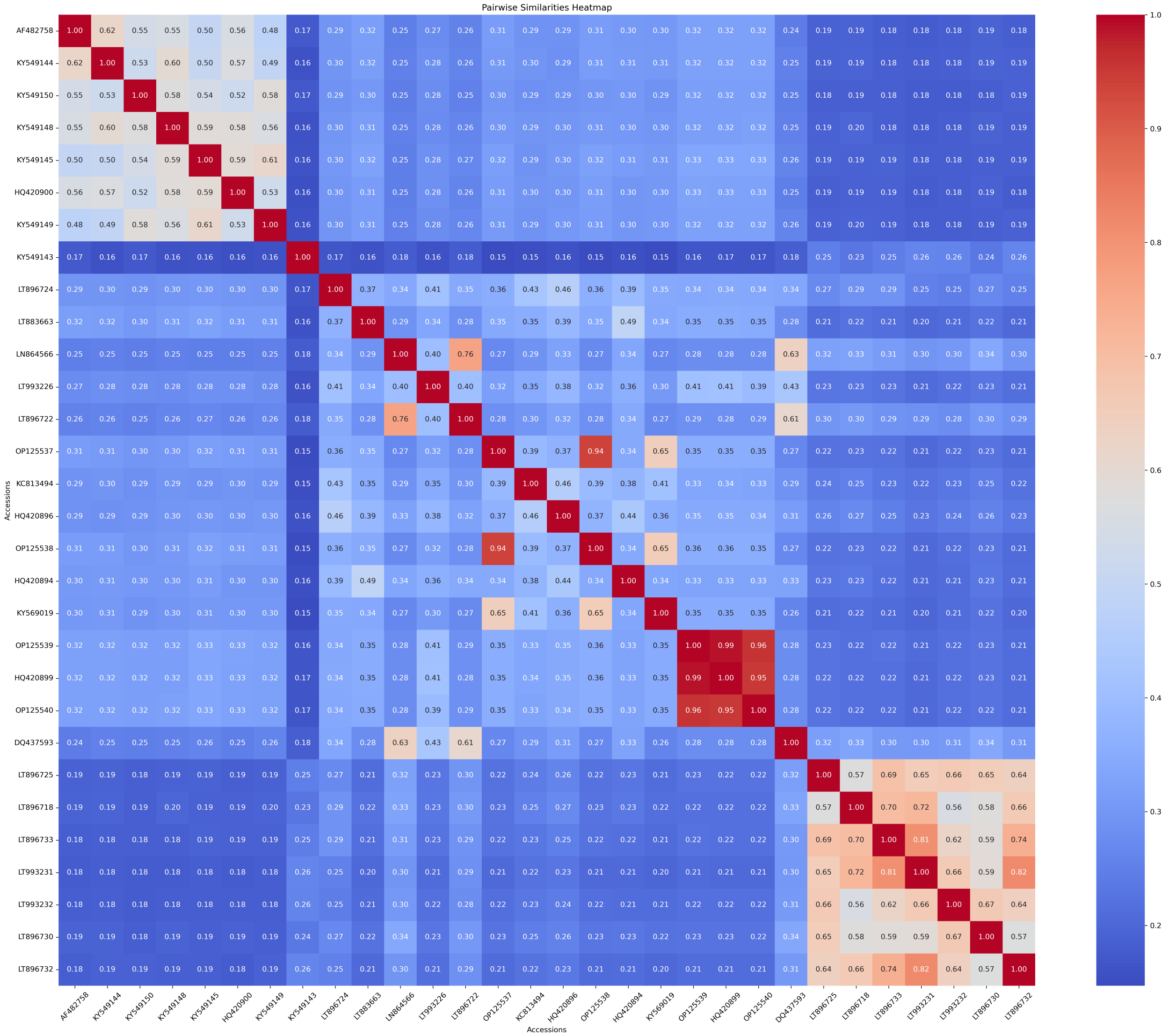

Figure S4: Bar plot showing the pairwise similarities between the higher scoring proteomes for the Cowpox Virus sample PXD014913. Similarities ranged between 0.1 and 1.

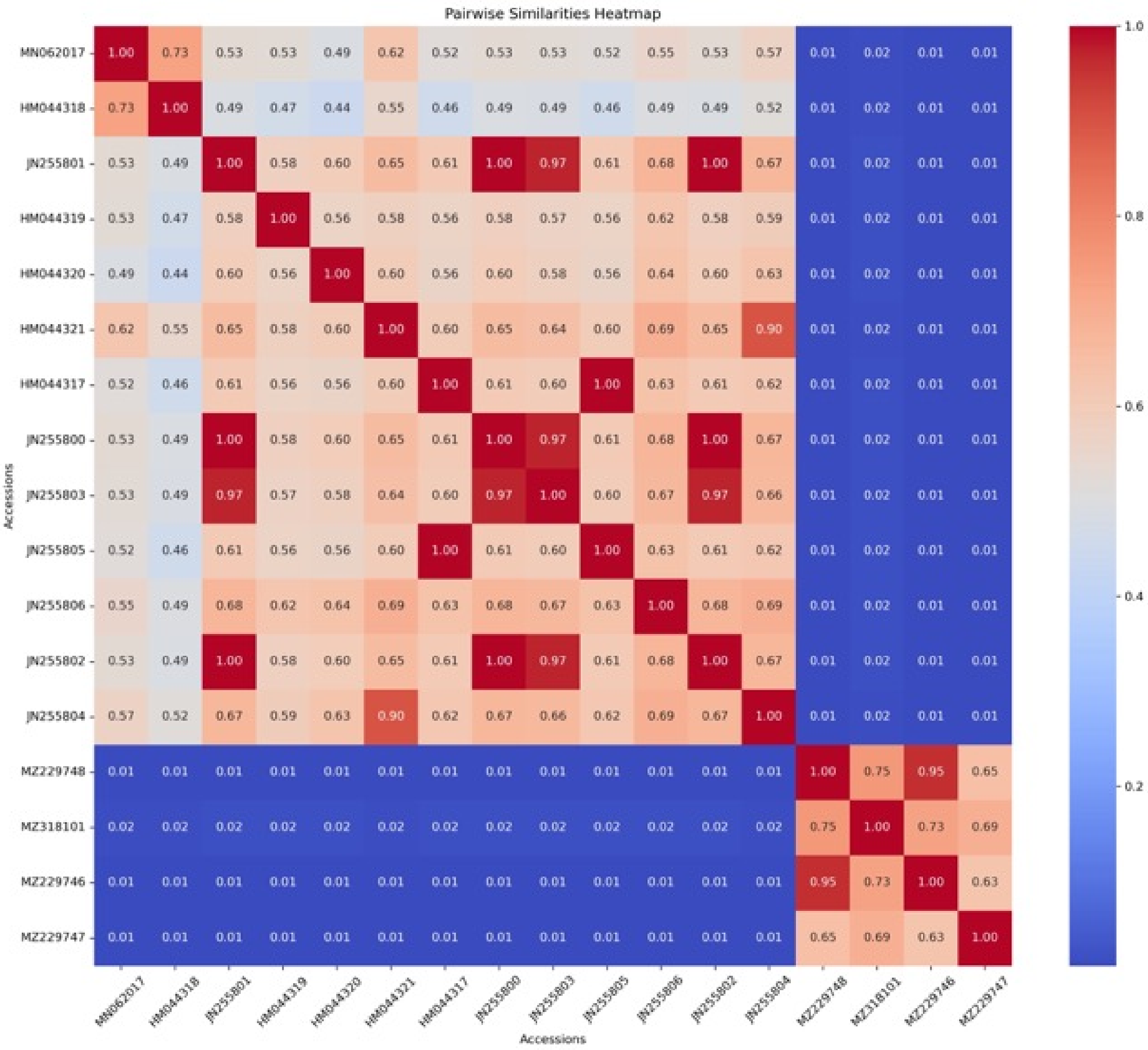

Figure S5: Bar plot showing the pairwise similarities between the higher scoring proteomes for the Hendra Virus. The lowest similarity score is 0.6, as all included proteomes show high overlap. The lowest similarity is 0.01, but the upper right section in reddish colors shows that a large part of strains had a peptidome overlap of 0.6 or higher.

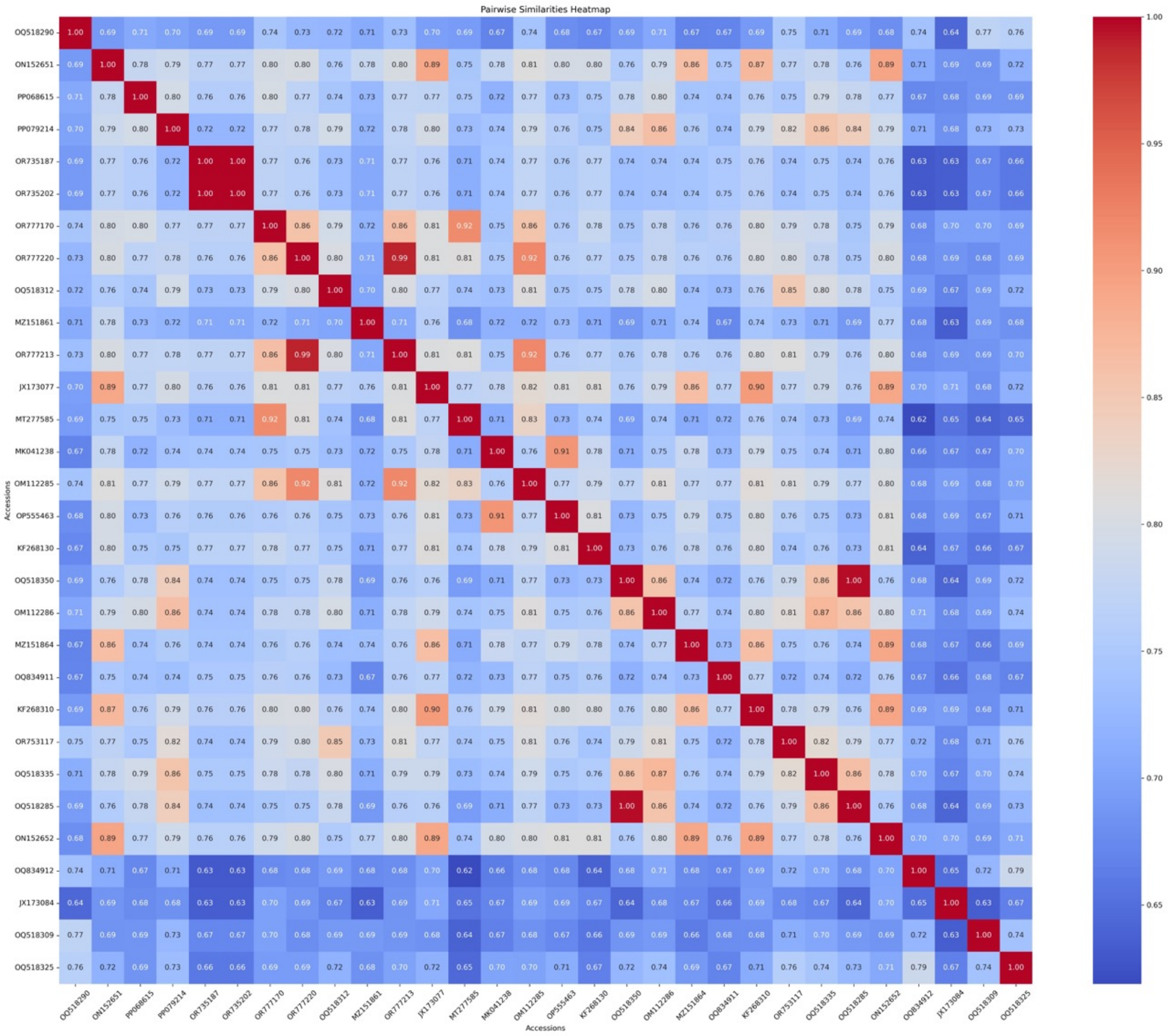

Figure S6: Bar plot showing the pairwise similarities between the higher scoring proteomes for the Adenovirus 2 . All genomes were quite similar, the lowest pairwise similarity displayed being 0.6.

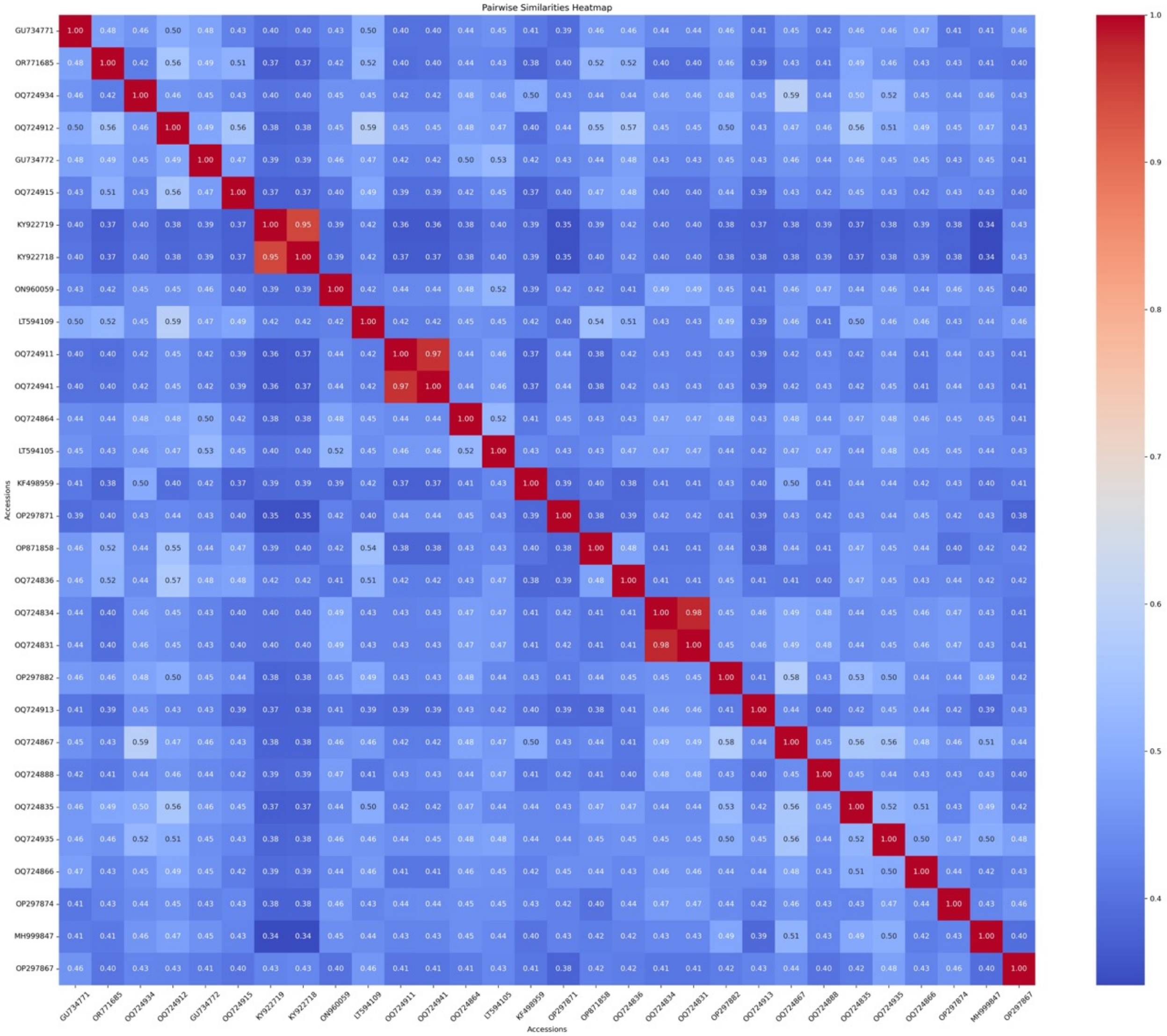

Figure S7: Bar plot showing the pairwise similarities between the higher scoring proteomes for the herpes simplex virus. The lowest similarity score is 0.5, as alle included proteomes show high overlap.

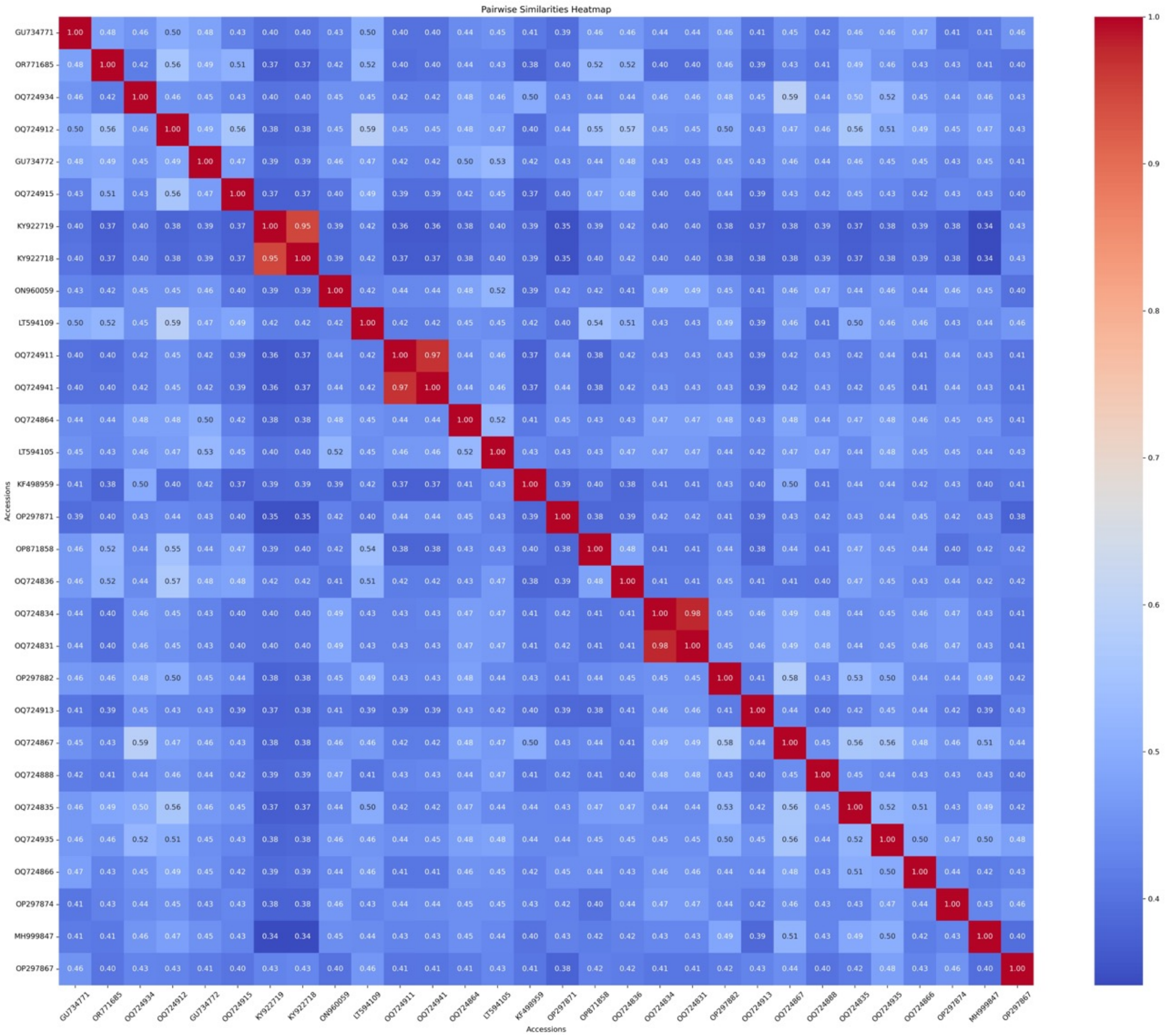

Figure S8: Bar plot showing the pairwise similarities between the higher scoring proteomes for the Mastadeno virus. The lowest similarity score is 0.75, but most peptidome have an overlap of more than 0.95.

COVID Mode

The COVID mode (Figure S9) is automatically activated and interposed when SARS-CoV-2 is identified during the reference database search. In this mode, a specialized query approach is used to refine candidate sequences before proceeding with further analyses.

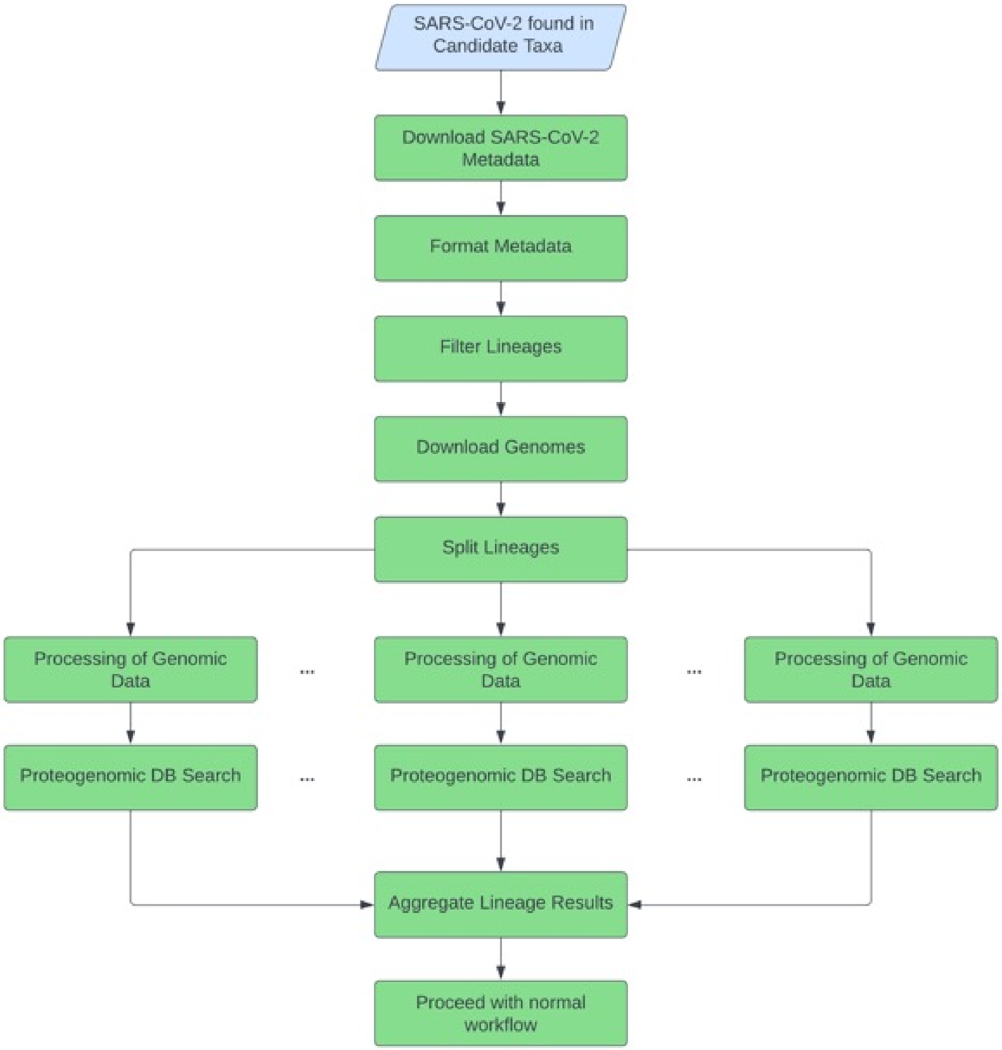

Figure S9: **Flowchart showing the workflow for the COVID mode.**
The blue box represents the input. Green boxes show the workflow of this mode. For each lineage cluster, the steps "Processing of Genomic Data" and "Database Search" are performed.

Selection of SARS-CoV-2 lineages

To identify relevant SARS-CoV-2 sequences, we use the NCBI datasets-CLI tool[5] to inspect metadata from genomic sequence entries available in the NCBI database and to determine, which genomic sequences should be included for the following database search step. Only sequences labeled as *complete* are considered for downstream processing to ensure high data quality.

Since a comprehensive database search encompassing all SARS-CoV-2 lineages and sub-lineages is computationally impractical—due to both the vast dataset size and the high sequence similarity of closely related sub-lineages—we apply a redundancy reduction strategy. Specifically, we include only a subset of sub-lineages, ensuring that selected representatives maintain an accuracy threshold ± 1 sub-levels within the lineage hierarchy.

Sequence retrieval and lineage clustering

To retrieve nucleotide sequences, MultiStageSearch performs an Entrez query using the GenBank accession identifiers extracted from the metadata. Given the high redundancy within SARS-CoV-2 lineage data, we further refine our approach by making use of the hierarchical nomenclature of SARS-CoV-2 lineages.[21] Lineage relationships are based on ancestors and descendants, where each lineage is recognized as a descendant of its direct ancestor (e.g., lineage B.1 is a descendant of B). Furthermore, lineages with three or more points in their name are assigned an alias to improve readability and clearness of the nomenclature. We only include a subset of these lineages, leaving out lineages with with two and three points. To enhance efficiency of our approach, lineages are clustered based on shared alphabetical prefixes in their designation (e.g., clusters such as AY and B). If a cluster does not meet the user-defined threshold for minimum lineage representation, its members are reassigned to a generic "others" cluster.

Integration with the main workflow

For each cluster, genomic data is processed and analyzed following the procedures outlined in the sections "Generation of the Proteogenomic Reference Database" and "Genomic DB Search". After processing all clusters, lineage-specific results are aggregated, and the top-scoring lineages are determined. These selected lineages are then fed back into the genomic database search within the main workflow, allowing the standard pipeline to continue from this intermediate step.

Evaluation of the COVID mode

The COVID mode is able to capture the full depth of SARS-CoV-2 lineages with an accuracy of ± 1 sub-levels. This accuracy is achieved due to the nomenclature-based alias assignment, which ensures that lineages with more than three hierarchical levels receive a designated alias.

A sub-lineage with one point (e.g., B.1) covers the first two sub-levels and the third sub-level (e.g., B.1.1.1) is covered by the alias assigned for the fourth sub-level (C.1 is the alias for B.1.1.1.1). In the case of the first two sub-levels, this is covered by the lineages with one dot while the third sub-level is covered by the new alias assigned by the nomenclature.

When using COVID mode, MultiStageSearch successfully infers the correct lineage for the PXD025130 SARS-CoV-2 sample with the aforementioned accuracy. As shown in Figure S10, the highest weight is attributed to "B.60", which is a direct descendant of the correct lineage "B". In contrast, MultiStageSearch fails to infer the correct lineage for the PXD018594_SARS-CoV-2 sample. As shown in Figure S11, while the correct lineage is "B", "W.1" has the highest weight. This alias corresponds to "B.1.177.53.1".

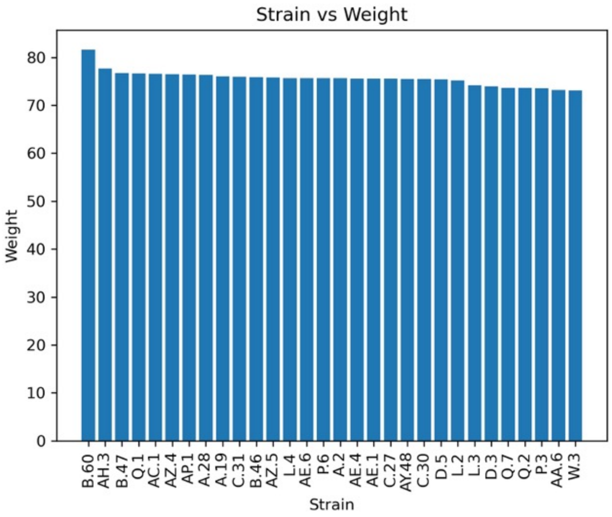

Figure S10: Results of MultiStageSearch using the Covid mode for the PXD025130_SARS-CoV-2 sample.

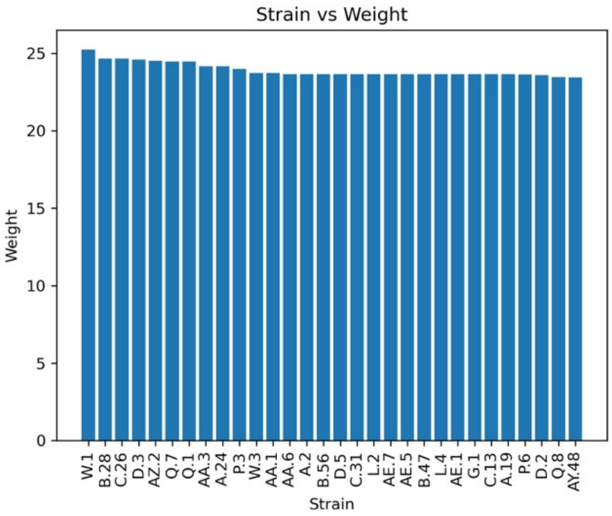

Figure S11: Results of MultiStageSearch using the Covid mode for the PXD018594_SARS-CoV-2 sample.

References

(1) Wes McKinney Data Structures for Statistical Computing in Python. Proceedings of the 9th Python in Science Conference. 2010; pp 56 – 61.

(2) Chapman, B.; Chang, J. Biopython: Python tools for computational biology. *ACM Sigbio Newsletter* **2000**, *20*, 15–19.

(3) Harris, C. R.; Millman, K. J.; Van Der Walt, S. J.; Gommers, R.; Virtanen, P.; Cournapeau, D.; Wieser, E.; Taylor, J.; Berg, S.; Smith, N. J.; others Array programming with NumPy. *Nature* **2020**, *585*, 357–362.

(4) Huerta-Cepas, J.; Serra, F.; Bork, P. ETE 3: reconstruction, analysis, and visualization of phylogenomic data. *Molecular biology and evolution* **2016**, *33*, 1635–1638.

(5) Sayers, E. W.; Beck, J.; Bolton, E. E.; Bourexis, D.; Brister, J. R.; Canese, K.; Comeau, D. C.; Funk, K.; Kim, S.; Klimke, W.; others Database resources of the national center for biotechnology information. *Nucleic acids research* **2021**, *49*, D10.

(6) Nguyen, L.-T.; Schmidt, H. A.; Von Haeseler, A.; Minh, B. Q. IQ-TREE: a fast and effective stochastic algorithm for estimating maximum-likelihood phylogenies. *Molecular biology and evolution* **2015**, *32*, 268–274.

(7) Katoh, K.; Toh, H. Recent developments in the MAFFT multiple sequence alignment program. *Briefings in bioinformatics* **2008**, *9*, 286–298.

(8) Python Package Index - PyPI. `https://pypi.org/`.

(9) Craig, R.; Beavis, R. C. TANDEM: matching proteins with tandem mass spectra. *Bioinformatics* **2004**, *20*, 1466–1467.

(10) Käll, L.; Canterbury, J. D.; Weston, J.; Noble, W. S.; MacCoss, M. J. Semi-supervised learning for peptide identification from shotgun proteomics datasets. *Nature methods* **2007**, *4*, 923–925.

(11) Gabriels, R.; Declercq, A.; Bouwmeester, R.; Degroeve, S.; Martens, L. psm_utils: A high-level python API for parsing and handling peptide-spectrum matches and proteomics search results. *Journal of Proteome Research* **2022**, *22*, 557–560.

(12) Buur, L. M.; Declercq, A.; Strobl, M.; Bouwmeester, R.; Degroeve, S.; Martens, L.; Dorfer, V.; Gabriels, R. MS^{2}Rescore 3.0 is a modular, flexible, and user-friendly platform to boost peptide identifications, as showcased with MS Amanda 3.0. **2024**,

(13) Hunter, J. D. Matplotlib: A 2D graphics environment. *Computing in science & engineering* **2007**, *9*, 90–95.

(14) Waskom, M. L. seaborn: statistical data visualization. *Journal of Open Source Software* **2021**, *6*, 3021.

(15) Rice, P.; Longden, I.; Bleasby, A. EMBOSS: the European molecular biology open software suite. *Trends in genetics* **2000**, *16*, 276–277.

(16) R Core Team R: A Language and Environment for Statistical Computing. R Foundation for Statistical Computing: Vienna, Austria, 2021.

(17) Wickham, H.; Hester, J.; Bryan, J. readr: Read Rectangular Text Data. 2024; R package version 2.1.5.

(18) Xie, Y. *Dynamic Documents with R and knitr*; Chapman and Hall/CRC, 2017.

(19) Wickham, H.; François, R.; Henry, L.; Müller, K.; Vaughan, D. dplyr: A Grammar of Data Manipulation. 2023; R package version 1.1.4.

(20) Xie, Y.; Cheng, J.; Tan, X. DT: A Wrapper of the JavaScript Library 'DataTables'. 2024; R package version 0.33.

(21) Rambaut, A.; Holmes, E. C.; O'Toole, Á.; Hill, V.; McCrone, J. T.; Ruis, C.; du Plessis, L.; Pybus, O. G. A dynamic nomenclature proposal for SARS-CoV-2 lineages to assist genomic epidemiology. *Nature microbiology* **2020**, *5*, 1403–1407.
